# Discovery of novel variants in genotyping arrays improves genotype retention and reduces ascertainment bias

**DOI:** 10.1186/1471-2164-13-34

**Published:** 2012-01-19

**Authors:** John P Didion, Hyuna Yang, Keith Sheppard, Chen-Ping Fu, Leonard McMillan, Fernando Pardo-Manuel de Villena, Gary A Churchill

**Affiliations:** 1 Department of Genetics, University of North Carolina at Chapel Hill, CB 7264, Chapel Hill, North Carolina, 27599-7264, USA; 2 Lineberger Comprehensive Cancer Center, University of North Carolina at Chapel Hill, CB 7295, Chapel Hill, North Carolina, 27599-7295, USA; 3 Carolina Center for Genome Science, University of North Carolina at Chapel Hill, CB 7264, Chapel Hill, North Carolina, 27599-7264, USA; 4 Department of Biostatistics and Bioinformatics, Duke University Medical Center, Box 2721, Durham, NC, 27710, USA; 5 Center for Genome Dynamics, The Jackson Laboratory, 600 Main Street, Bar Harbor, Maine, 04609, USA; 6 Department of Computer Science, University of North Carolina at Chapel Hill, CB 3175, Chapel Hill, North Carolina, 27599-3175, USA

## Abstract

**Background:**

High-density genotyping arrays that measure hybridization of genomic DNA fragments to allele-specific oligonucleotide probes are widely used to genotype single nucleotide polymorphisms (SNPs) in genetic studies, including human genome-wide association studies. Hybridization intensities are converted to genotype calls by clustering algorithms that assign each sample to a genotype class at each SNP. Data for SNP probes that do not conform to the expected pattern of clustering are often discarded, contributing to ascertainment bias and resulting in lost information - as much as 50% in a recent genome-wide association study in dogs.

**Results:**

We identified atypical patterns of hybridization intensities that were highly reproducible and demonstrated that these patterns represent genetic variants that were not accounted for in the design of the array platform. We characterized variable intensity oligonucleotide (VINO) probes that display such patterns and are found in all hybridization-based genotyping platforms, including those developed for human, dog, cattle, and mouse. When recognized and properly interpreted, VINOs recovered a substantial fraction of discarded probes and counteracted SNP ascertainment bias. We developed software (MouseDivGeno) that identifies VINOs and improves the accuracy of genotype calling. MouseDivGeno produced highly concordant genotype calls when compared with other methods but it uniquely identified more than 786000 VINOs in 351 mouse samples. We used whole-genome sequence from 14 mouse strains to confirm the presence of novel variants explaining 28000 VINOs in those strains. We also identified VINOs in human HapMap 3 samples, many of which were specific to an African population. Incorporating VINOs in phylogenetic analyses substantially improved the accuracy of a *Mus *species tree and local haplotype assignment in laboratory mouse strains.

**Conclusion:**

The problems of ascertainment bias and missing information due to genotyping errors are widely recognized as limiting factors in genetic studies. We have conducted the first formal analysis of the effect of novel variants on genotyping arrays, and we have shown that these variants account for a large portion of miscalled and uncalled genotypes. Genetic studies will benefit from substantial improvements in the accuracy of their results by incorporating VINOs in their analyses.

## Background

Hybridization arrays are widely used in research, clinical, and commercial applications involving humans, mice and other organisms to genotype single nucleotide polymorphisms (SNPs). Software is used to infer discrete genotypes using continuous intensity data from bi-allelic probes. However, existing methods are imperfect leading to incorrect calls and uncalled genotypes ("no-calls").

The Mouse Diversity Array [1] is a high-density genotyping platform similar to the Affymetrix Genome-Wide Human SNP 6.0 array. It contains probes for 623124 SNPs and 916269 invariant genomic probes (IGP) designed to broadly sample diversity within the *Mus musculus *species. SNP probes occur in sets of eight: two forward-strand and two reverse-strand probes for both an A allele, which corresponds to the reference sequence, and a B allele, which corresponds to the known variant. Opposite-strand probes are potentially offset by up to 10 bp. IGP probes occur in pairs, with one probe targeting each strand, and are not offset. The Mouse Diversity Array and similar platforms use genome-wide sampling to reduce genomic complexity by size-selective amplification of restriction fragments [2]. Efficient hybridization requires genomic DNA targeted by a probe set to fall within at least one restriction enzyme fragment in the selected size range (50 bp to 1 kb). The Mouse Diversity Array was designed to use a combination of two restriction enzymes, *NspI *and *StyI*, and fragment sizes were predicted based on the mouse reference genome (NCBI mouse genome Build 36).

Genotype calling programs use a variety of methods to infer discrete genotypes from continuous intensity data. Many methods, including the BRLMM-P algorithm developed by Affymetrix [3], employ clustering of multiple samples based on the contrast between allelic probe intensities. Samples belonging to the two clusters with a large absolute contrast are called as homozygous genotypes and samples with low contrast are called heterozygous. Samples that do not fall within any of the three clusters in the contrast dimension remain uncalled.

In an earlier study we genotyped 162 laboratory mouse strains using the Mouse Diversity Array [4]. We used these data to determine the subspecific origin and haplotype diversity of the laboratory mouse. As a model organism, the laboratory mouse has several distinct features that are key for this study. Laboratory strains sample the genetic variation present in genetically divergent species and subspecies. Most strains are fully homozygous as a result of dozens to hundreds of generations of inbreeding. F1 hybrids obtained by crossing two inbred strains have genotypes that can be accurately predicted from the parental genotypes. Finally, the mouse has a whole-genome reference sequence based on a single inbred strain (C57BL/6J) and 17 additional strains have been sequenced recently as part of the Sanger Institute's Mouse Genomes sequencing project (henceforth referred to as the Sanger strains) [5].

Contrary to our expectation of homozygosity at all SNPs in inbred mouse strains, we observed a substantial number of heterozygous genotype calls [1,4]. Furthermore, the rates of both no-calls and unexpected heterozygous calls were positively correlated with divergence from the reference genome. The highest rates were observed in strains derived from species of the *Mus *genus other than *Mus musculus*, such as *M. spretus *and *M. spicilegus *[1], followed by strains derived from the *M. m. musculus *and *M. m. castaneus *subspecies (whereas the C57BL/6J genome is primarily *M. m. domesticus *in origin). These findings illuminate problems affecting all hybridization arrays, genotype calling software and studies that use these genotype data for a variety of goals. Our studies of well-characterized inbred strains have brought these issues to the forefront and provide an opportunity for investigating the underlying causes of genotyping errors.

At any given time, only a subset of the genetic variation within a species is known. This creates a bias in SNPs available for array designs in favor of variants present in the best-studied individuals, populations or clades [6]. Furthermore, many arrays are designed using an iterative process that selects only probes that perform well across a screening set of samples. This is done to ensure low miscall and no-call rates, but can introduce further bias. Miscall and no-call rates can vary greatly depending on the composition of samples. As we have observed, and as noted in other studies [7], miscall and no-call rates are positively correlated with genetic divergence from the reference sequence used to design the array. Furthermore, when SNP probes are excluded from analyses due to post-hoc filtering based on no-call rate, unexpected heterozygosity or departure from Hardy-Weinberg equilibrium, important information is lost (discussed below) in addition to the introduction of further bias. In a recent genome-wide analysis of a large number of dog breeds, over 50% of SNPs were excluded for such reasons [8]. The cumulative effect of these SNP selection procedures can potentially skew the interpretation of experimental results and limit researchers' ability to effectively study genetically divergent samples. The Mouse Diversity Array was designed with attention to the phylogenetic origin of SNPs [1], but SNP selection will still introduce some biases, especially in studies that include wild-derived strains or wild-caught mice [4].

Essentially, a no-call or incorrect genotype call is the result of abnormal hybridization intensity for a sample at a given SNP and may be due to technical or biological causes. Technical issues, such as array manufacturing or DNA processing, would result either in systematic errors that affect all samples at that SNP (such as an incorrect probe sequence on the array) or all SNPs from a single sample (such as incomplete digestion). Errors of this class should be detectable. In addition, non-systematic stochastic errors may affect a small subset of genotype calls.

An additional source of genotype calling errors is biological in origin and can be attributed to previously uncharacterized variation in genomic DNA, either in the sequence targeted by a probe set or in the proximal or distal restriction sites used for genome-wide amplification. These variants can reduce hybridization intensity sufficiently to eliminate or reverse the contrast between allelic probes such that an incorrect genotype call (or no-call) is made. We term such variants "off-target variants" (OTVs) to distinguish them from the expected variant targeted by the SNP probe set. We term probe sets affected by OTVs as variable intensity oligonucleotides (VINOs) due to the dynamic effect of OTVs on hybridization intensity.

Genotyping errors due to uncharacterized sequence variation have been observed in microsatellite genotyping (termed "null alleles") and were recently subjected to systematic analysis [9,10], however they have gone largely unaddressed in SNP genotyping studies. In this study, we investigated the effect and extent of OTVs in a diverse collection of inbred strains and intercrossed mice using the Mouse Diversity Array. We conclude that OTVs are the primary cause of miscalls and no-calls. Furthermore, we determined that a substantial fraction of VINOs can be reliably identified, and we have developed MouseDivGeno [11], a novel genotype-calling algorithm implemented as a package for the R language [12]. We demonstrate the accuracy of our algorithm by comparison with other genotype calling software and with whole-genome sequence of the Sanger strains. The ability to recognize VINOs and treat samples having OTVs as a distinct genotype class will enable SNP discovery, increase the power of evolutionary and association studies, and lend itself to potential clinical applications. Finally, we investigated the extent of off-target variation and its effect on genotype calls. Our findings suggest ways to improve both array design and genotype calling algorithms.

## Results and discussion

### Identification of VINOs

We hybridized 351 mouse DNA samples on the Mouse Diversity Array (additional file 1). We have made the raw data available in the Affymetrix CEL format [11]. This sample set included classical inbred strains, wild-derived strains with varying degrees of inbreeding, consomic strains, recombinant inbred strains, samples from early generations of the Collaborative Cross [13], F1 hybrids created by crossing classical and/or wild-derived strains and wild mice [4]. Among the 143 inbred strains in this sample (116 classical and 27 wild-derived), we observed a significant increase in both heterozygous calls and no-calls as a function of genetic distance from the reference genome (additional file 2). All of these strains are expected to be fully homozygous based on previous studies (for at least 99% of their genomes) [4], therefore we assume that most of the heterozygous calls are errors (miscalls).

**Figure 1:**
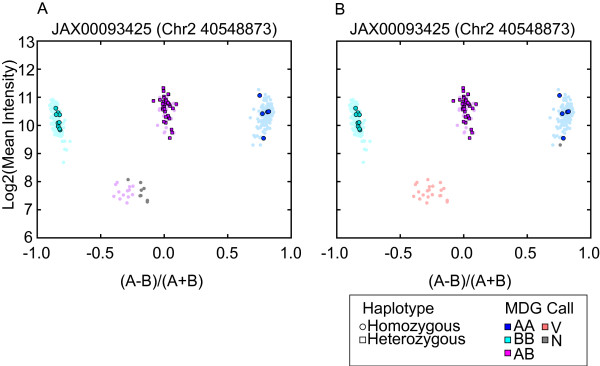
**MouseDivGeno identifies VINOs as a cluster of low-intensity samples**. Contrast plots of a SNP called by A) BRLMM-P 2D and B) MouseDivGeno. Probe intensities from 351 samples (additional file 1) are shown in MA-transformed space. The sample contrast is the normalized difference between A and B allele intensities [(A-B)/(A+B)]. The y-axis shows the log2 mean of A and B allele intensities. Dark blue: AA call; light blue: BB call; purple: AB call; red: V call; gray: N call. Circles represent strains with a homozygous haplotype in the region of the SNP, while squares represent strains with a heterozygous haplotype. F1 animals with parental alleles of AA and BB are true heterozygotes and are highlighted along with their parental strains. MouseDivGeno software is able to identify samples in the low intensity cluster as containing an OTV and assigns a VINO (V) call, whereas BRLMM-P 2D assigns several different genotype calls (AB, N) to samples in this cluster.

To further characterize these putative genotyping errors, we developed a tool for visualization of probe set hybridization intensities in which intensity contrast is plotted against average intensity [14]. These plots revealed that most heterozygous calls in inbred strains are easily distinguished from true heterozygous calls by greatly reduced average intensity (Figure 1). These initial findings suggested that genotyping algorithms that discriminate based only on contrast (such as BRLMM-P) would be unable to detect VINOs.

The term VINO refers to a probe set that displays a low-intensity cluster such as the one shown in Figure 1,and it applies to a probe set in the context of the collection of samples being genotyped. Identification of VINOs can be hampered by several factors including the underrepresentation of genotype classes in a sample set, batch effects, and probe-sequence-specific effects that increase intensity variability within genotype groups. We have applied a stringent set of criteria to detect VINOs but, as discussed below, the impact of OTVs is not limited to only those probe sets that are declared to be VINOs.

### VINOs indicate the presence of OTVs

Manual inspection of intensity data led to the identification of multiple VINOs. To test whether previously unknown genetic variants were present in the target genomic DNA of these probe sets, we sequenced genomic regions surrounding 15 SNPs in different subsets of 72 strains selected to ensure that each genotype class would be present in a minimum of four samples (see METHODS, additional file 3). Of these, 14 probe sequences contained at least one to as many as three OTVs among the strains sequenced. The single probe that did not contain any OTVs (JAX00303026) was notable due to the presence of a SNP in each of its distal *NspI *and *StyI *restriction sites and, as a result, both fragments fell outside of the optimal size range for whole genome amplification. We have also observed that a restriction fragment length polymorphism (RFLP) in only one of the flanking cut sites can reduce hybridization efficiency. The effect may be negligible or substantial depending on the size of the other fragment. Thus OTVs in the probe sequence or RFLPs in the flanking restriction sites explain all of the VINOs in this small set.

### Mouse Diversity Genotyping Software

The discovery, prevalence and distribution of VINOs among our sample set motivated us to create MouseDivGeno [11], an implementation of our genotype-calling algorithm as an R package. MouseDivGeno uses a novel combination of Gaussian mixture modeling and hierarchical clustering followed by a VINO detection step (details in METHODS). We used MouseDivGeno to genotype the 351 samples (additional file 1) for 526363 autosomal SNP probe sets that were above thresholds based on performance indices. MouseDivGeno produces two sets of calls: genotypes, which include standard calls for homozygous (AA and BB), heterozygous (AB) and no-calls (N); and VINOtypes, which substitute VINO (V) calls for standard genotype calls when the VINO criteria is satisfied (METHODS). In the 351 samples set, MouseDivGeno made 786274 VINO calls (0.43%).

**Figure 2:**
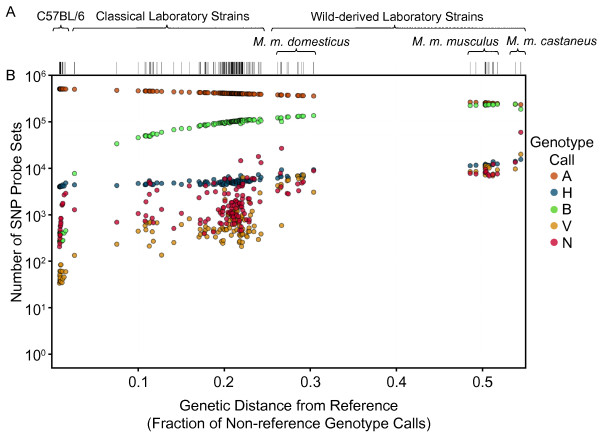
**Non-homozygous VINOtype call rates increase with divergence from the reference genome**. A) Genetic distance from the mouse reference genome for 143 laboratory inbred strains (additional file 1). Each strain is shown as a vertical tick mark. Strains are grouped according to their origin are arranged left-to-right in increasing order of genetic distance from the reference. Genetic distance is computed as the fraction of non-reference (non-A allele) genotype calls. B) VINOtype calls for each strain. For each strain, the number of SNP probe sets assigned each of the five possible calls (A, B, H, V or N) are shown as five points of different colors that sum to 526363 SNP probe sets. The same analysis using only standard genotype calls (A, B, H, N) is shown in additional file 2.

Among the 143 inbred samples, the rate of VINOs is 0.35% with a per-sample range from 34 to 20398 (0.006-3.88%). The largest fraction of VINO calls were originally genotyped as AB (47.1%), followed by AA, BB and N calls (33.1%, 15.3% and 4.5%, respectively). However, within each genotype class, N calls were most commonly converted to VINO calls (28.4%), followed by AB, BB and AA calls (6.43%, 0.065% and 0.006%, respectively). Only a fraction of heterozygous calls are converted to VINOs, which explains why the positive correlation between genetic distance and miscalls and no-calls remains even after VINOtyping (Figure 2). The VINOtyping algorithm is intentionally calibrated to be conservative in order to minimize the loss of correct genotype calls.

**Figure 3:**
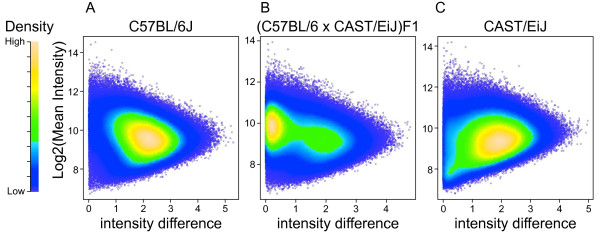
**Global intensity patterns discriminate between classical inbred, genetically divergent and hybrid samples**. Hybridization intensity for all SNP probes for A) C57BL6/J, a classical inbred strain and also the mouse reference genome, C) CAST/EiJ, an inbred strain derived from a wild-caught *M. m. castaneus *mouse, and B) a (C57BL/6JxCAST/EiJ)F1 hybrid. The x-axis represents the absolute value of the contrast (A-B) and the y-axis is the mean intensity. The density of probe sets is represented by a color gradient from purple (low) to green (high). VINOs are seen in the lower-left corner of each plot.

The reference strain C57BL/6J has only a small number of VINO calls. Examination of the global distribution of the intensity-contrast values (Figure 3) confirms that there are few probes with low contrast values and low mean intensity (lower left region of Figure 3A). CAST/EiJ (a strain derived from *M. m. castaneus*) has a large number of VINOs and there is a corresponding high density of probes in the lower left region of Figure 3C. The majority of probes for both of these two inbred strains have high contrast values reflecting the predominance of homozygous genotypes. On the other hand the F1 hybrid (Figure 3B) has a large proportion of low contrast probes indicating high levels of heterozygosity. The VINOs that are apparent in the CAST/EiJ strain are "rescued" by the C57BL/6J allele in the F1 hybrid and many of them appear as an incorrect homozygous call with moderate mean intensity but high contrast values.

### MouseDivGeno genotyping is highly concordant with existing methods and with SNP discovery by whole genome sequencing

We compared MouseDivGeno genotype calls with two other methods: ALCHEMY [15] and BRLMM-P 2D [3] (Table 1). Genotype calls for the set of 351 samples were highly concordant in homozygous (AA, BB) and heterozygous (AB) classes (97.4%, 97.8% and 97.8% agreement for MouseDivGeno/ALCHEMY, MouseDivGeno/BRLMM-P 2D and ALCHEMY/BRLMM-P 2D two-way comparisons, respectively). The majority of discordant genotypes were due to homozygous calls using one of the methods that were called heterozygous using another method. Conflicts with opposite homozygous genotypes were very rare (less than 0.05% in all comparisons). The overall rate of AB genotypes was slightly lower for MouseDivGeno (10.26%) compared to ALCHEMY (11.45%) and BRLMM-P 2D (11.62%). Of the VINO calls from MouseDivGeno, 9.76% and 46.04% were called AB by ALCHEMY and BRLMM-P 2D, respectively, while 65.32% and 34.04% were called as N.

**Table 1:** Pairwise concordance between genotyping methods

	Alchemy			
**MouseDivGeno**	**AA**	**AB**	**BB**	**N**

AA	127,867,623	1,920,909	25,627	1,026,558
AB	769,150	16,433,765	635,019	1,116,810
BB	3,425	1,257,523	29,282,297	560,353
NN	931,375	1,458,539	277,201	400,965
V	132,229	76,720	63,696	513,629
	**BRLMM-P 2D**			

**MouseDivGeno**	**AA**	**AB**	**BB**	**N**

AA	129,030,882	824,258	20,829	964,748
AB	1,089,922	17,190,764	335,325	338,733
BB	27,880	1,628,799	29,044,569	402,350
NN	1,037,029	1,469,896	258,170	302,985
V	104,122	361,969	52,523	267,660
	**BRLMM-P 2D**			

**Alchemy**	**AA**	**AB**	**BB**	**N**

AA	128,281,002	675,020	16,046	731,734
AB	1,865,751	18,131,854	388,169	761,682
BB	60,277	987,808	28,913,772	321,983
NN	1,082,805	1,681,004	393,429	461,077

Of the Sanger strains, 14 are *M. musculus *inbred strains that were genotyped with the Mouse Diversity Array. We obtained and filtered SNPs and small insertions/deletions (indels) at autosomal typed loci (METHODS). As expected due to the inbred status of these 14 strains, there are no heterozygous calls in the filtered Sanger genotypes. Heterozygous call rates among these 14 samples were 1.25%, 1.99% and 1.25% for MouseDivGeno, ALCHEMY and BRLMM-P 2D, respectively (Table 2). Among homozygous SNP calls, we observed 99.8% concordance between each of the three array-based methods and the Sanger genotypes. MouseDivGeno made 35604 VINO calls (0.48% of total calls), a proportion similar to the one observed in the larger set of 351 samples. Among VINOs, 81.4% correspond to an AA or BB homozygous genotype calls in the Sanger data. Because Sanger SNPs were identified by alignment to the reference sequence, regions that could not be aligned were inaccessible to SNP discovery and thus not comparable with array genotypes. As expected, the inaccessible fraction of the genome increases with a strain's divergence from the reference. We observed an enrichment of VINO calls in inaccessible regions of the Sanger data (2221 VINO calls compared to an expectation of 54) [5], in probes with a deleted target base (24 vs. 2 expected) and unaligned or non-uniquely aligned probes (4361 vs. 82 expected).

**Table 2:** Pairwise concordance of genotype calls between Sanger and array-based methods

	Sanger					
**MouseDivGeno**	**AA**	**BB**	**Deleted**	**Inaccessible**	**Excluded**	**Total**

**AA**	5,488,489	5,327	88	4,818	3,504	5,502,226
**AB**	51,460	39,080	24	424	1,316	92,304
**BB**	7,244	1,627,823	181	3,022	6,230	1,644,500
**V**	13,510	15,488	24	2,221	4,361	35,604
**N**	43,605	48,659	19	577	1,588	94,448
**Total**	5,604,308	1,736,377	336	11,062	16,999	7,369,082

**Alchemy**	**AA**	**BB**	**Deleted**	**Inaccessible**	**Excluded**	**Total**

**AA**	5,441,864	3,604	87	4,612	3,262	5,453,429
**AB**	97,883	46,767	34	553	1,741	146,978
**BB**	6,582	1,644,208	141	2,231	4,314	1,657,476
**N**	57,979	41,798	74	3,666	7,682	111,199
**Total**	5,604,308	1,736,377	336	11,062	16,999	7,369,082

**BRLMM-P 2D**	**AA**	**BB**	**Deleted**	**Inaccessible**	**Excluded**	**Total**

**AA**	5,523,440	7,022	63	4,673	3,456	5,538,654
**AB**	30,970	54,075	70	2,097	4,839	92,051
**BB**	7,702	1,631,208	169	2,509	5,164	1,646,752
**N**	42,196	44,072	34	1,783	3,540	91,625
**Total**	5,604,308	1,736,377	336	11,062	16,999	7,369,082

### Location of OTVs determine effect on intensity

**Figure 4:**
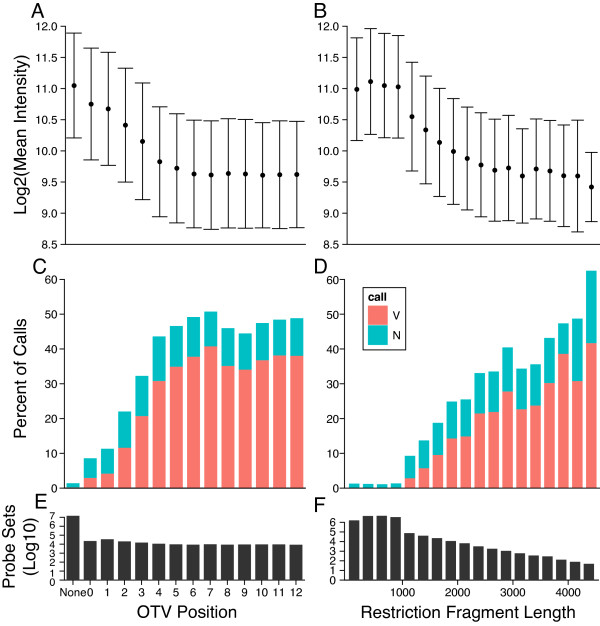
**OTV position in the probe and RFLP have significant effects on hybridization intensity and VINO detection**. Left panels: probe sets are grouped by the distance from the OTV to the nearest edge of the probe sequence for each possible OTV position (either none or between 0-12). Right panels: probe sets having no evidence of an OTV within the probe sequence are grouped by the size of their smallest restriction fragment (*NspI *or *StyI*) in bins of 250 bp. Top panels show the mean intensity across each subset using the four probes for the best-hybridizing allele in each probe set for A) OTV position in the probe and B) minimum restriction fragment length. Middle panels show the number of VINO and N calls (as a percentage of all genotype calls) for probe sets grouped by C) OTV position in the probe and D) minimum restriction fragment length. Bottom panels show the number of probes in each bin for E) OTV position in the probe and F) minimum restriction fragment length.

To identify the region of a probe in which we can reliably detect OTVs, we quantified the effect of OTVs on hybridization intensity (Figure 4A). We categorized SNP probes by whether they contained an OTV, and, if so, the offset of the OTV from the nearest end of the probe (from 0-12 bp). We then calculated the mean intensity of the probes for the best hybridizing allele for each probe set and plotted the distribution of intensities for each OTV position. We found that, as reported previously [16], OTVs located within the first 3 bp of either the 5' or 3' end of a target sequence (edge OTVs) had relatively minor effect on hybridization intensity. In contrast, OTVs within the central region of the probe (central OTVs) have pronounced effect on hybridization intensity, with mean intensity differing by more than one standard deviation from that of probes having no OTVs (Figure 4A).

We similarly examined the effect of RFLPs on intensity (Figure 4B) using probes with RFLP(s) that lead to suboptimal fragment size(s) but with no evidence of OTVs within the probe sequence. We found that mean intensity degrades as minimum fragment size increases above the maximum optimal size of 1 kb, and differs by more than one standard deviation from the mean of probe sets within the optimal size range when minimum fragment size increases above 1500 bp.

### Genomic features explain most VINOs predicted by MouseDivGeno

We identified all probe sets that had OTVs in the Sanger data. We excluded 16999 probe sets (0.23%) for which both strand probes were either unaligned or at least one strand probe was non-uniquely aligned (see METHODS). In the majority of these cases (92.26%), MouseDivGeno called a VINO or N. Of the remaining of probe sets, 4.12% had at least one event with the potential to disrupt hybridization (central OTV, edge OTV, RFLP or inaccessible). In more than half the cases where we observed one of these events, the event was present in both the forward and reverse strand probes, and MouseDivGeno was much more likely to call a VINO than when the event was present in only one strand probe (15.25% vs. 0.5%). MouseDivGeno detected VINOs at the highest rates in probe sets with central OTV(s) and low-coverage events (38.91% and 65.56%, respectively). When there was no evidence of an OTV, MouseDivGeno called a VINO at a rate of only 0.1% and made a concordant genotype call or no-call in most cases (97.6% and 1%, respectively).

We compared the rate of VINO calls to OTV position (Figure 4C) and minimum restriction fragment length (Figure 4D). We found that OTVs within the first or last 3 bp of the probe (edge OTVs) are less often recognized as VINOs compared to OTVs in the center region of the probe (central OTVs). MouseDivGeno called only 13.4% of all probes having an edge OTV as VINOs, compared with 45.0% of all probes having a central OTV. RFLPs alone (in the absence of any other OTV) have a smaller effect on intensity than do OTVs within a probe, and thus MouseDivGeno has a lower call rate (14.7% of probes with a minimum fragment size > 1 kb). There is a distinct threshold for RFLP length at 1.5 kb, above which we call VINOs at a substantially higher rate (23.7% vs. 10.9% for RFLP between 1-1.5 kb). In subsequent analyses we separated RFLPs into two categories based on this threshold.

We classified each VINO called by MouseDivGeno as having a central OTV, edge OTV, RFLP (1-1.5 kb or > 1.5 kb), within-probe cut site (a special case of RFLP in which an OTV introduces a new restriction site within the probe sequence), low sequencing coverage, multiple, or none of these features (additional file 4). There are 7073 VINO calls for which there is no high-confidence evidence of OTVs in the Sanger data and thus remain unexplained in our analysis. We expect that a significant number of these are due either to poorly performing probe-sets that were just above the threshold of exclusion from our analysis, incomplete SNP discovery in the Sanger strains, larger insertions or deletions or unresolved problems with the reference sequence assembly [17]. We examined the performance of ALCHEMY and BRLMM-P 2D and found a more than 30-fold increase in no-call rates for unexplained VINOs (48.5% and 38.7%, respectively, additional file 5).

**Figure 5:**
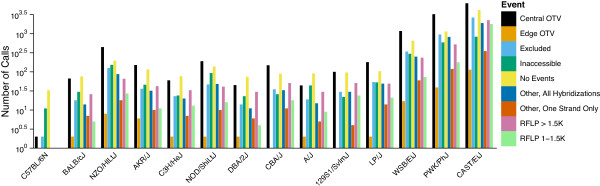
**VINO call rate and accuracy increases with divergence from the reference genome**. Breakdown of VINO calls in 14 Sanger strains (ordered from left to right by increasing divergence from the reference genome) by event type observed within the probe sequence. VINO calls with no corresponding evidence in the Sanger data (dark pink) comprise a larger proportion of all VINO calls for strains more similar to the reference than those that are more distant.

We broke down VINO calls by strain (Figure 5, additional file 6). The effects of genetic distance from the C57BL/6J reference genome are dramatic. At the time that the Mouse Diversity Array was designed, the reference sequence was available (NCBI build 36) but SNP discovery was conducted using only a handful of strains and with significant strain differences in the false negative rates [18,19]. This had a direct impact on our ability, at the time, to filter candidate SNPs for the Mouse Diversity Array, eliminating those with known OTVs. As result, 57.29% of VINOs were identified in CAST/EiJ and 21.41% in PWK/PhJ, the strains that are most genetically distinct from C57BL/6J. Similarly, the majority of OTVs are found in these strains.

### Effect of heterozygosity on VINO calls

**Figure 6:**
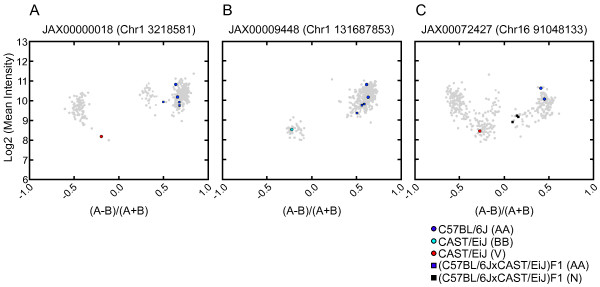
**Detected and undetected VINOs in homozygosity may lead to inaccurate genotyping in heterozygosity**. Circles represent parental strains: C57BL/6J (dark blue), which have the AA allele; CAST/EiJ, which has the BB genotype at its target position and also an OTV within the probe and is called either BB (light blue) or V (red) by MouseDivGeno; squares: (C57BL/6JxCAST/EiJ)F1 samples, which have an OTV in heterozygosity and are called AA (dark blue) or N (black) by MouseDivGeno. A) MouseDivGeno calls CAST/EiJ as V; the F1 samples are called AA due to stronger hybridization intensity for the AA allele and thus the OTV goes unrecognized. B) MouseDivGeno calls CAST/EiJ as BB due to the absence of a true BB cluster; the F1 samples are again called AA. C) MouseDivGeno calls CAST/EiJ as V but calls the F1 samples as N due to poor discrimination between genotype clusters.

To investigate the effect of OTVs in heterozygosity we analyzed F1 hybrid mice included in our 351 samples. These F1s were generated by crossing two inbred strains for which we should be able predict the genotypes at every locus in the hybrid based on the genotypes of the parental strains. Here we describe analysis of the (C57BL/6J × CAST/EiJ)F1 hybrid mouse (Figures 3 and 6, Table 3). Similar results were obtained on other F1 hybrid combinations (data not shown).

**Table 3:** Genotype calls for an F1 hybrid compared to its parental strains

		(C57BL/6JxCAST/EiJ)F1					
**C57BL/6J**	**CAST/EiJ**	**AA**	**BB**	**AB**	**V**	**N**	**Margin**

AA	AA	231,536	8	149	900	6,311	238,904
BB	BB	0	204	1	1	3	209
AA	BB	8,275	8,927	156,872	1,797	12,348	188,219
BB	AA	4	12	6	0	3	25
AA	V	12,605	19	1,681	1,578	4,445	20,328
BB	V	0	10	0	1	0	11
V	AA	4	0	0	1	0	5
V	BB	0	9	2	1	1	13
V	V	3	0	0	11	0	14
AA	AB	5,936	13	6,221	190	1,345	13,705
BB	AB	0	7	1	0	2	10
AB	AA	52	0	7	0	0	59
AB	BB	2	1,047	467	12	148	1,676
AB	AB	6	12	1,939	26	75	2,058
AA	N	27,921	3,193	22,039	508	6,650	60,311
BB	N	0	14	0	0	0	14
AB	N	4	106	136	5	27	278
V	N	0	4	0	0	0	4
N	N	36	3	13	0	2	54
N	AA	267	0	0	1	7	275
N	BB	3	5	116	0	16	140
N	AB	3	0	2	0	0	5
V	AB	0	0	0	1	0	1
AB	V	0	13	16	6	3	38
N	V	4	0	0	2	1	7
		286,661	13,606	189,668	5,041	31,387	526,363

OTVs in heterozygosity in the F1 hybrid are preferentially (62%) genotyped by MouseDivGeno as homozygous for the allele present in the non-VINO carrying parental strain (Figure 6A, Table 3). This result is expected based on the same principles that determine the behavior of VINOs in homozygosity. A parental strain with an OTV causing a small reduction in hybridization that does not lead to a VINO call may have an offspring with a true heterozygous genotype that is incorrectly called homozygous for the non-OTV allele. For example, the maternal strain has an AA genotype and the paternal strain has a BB genotype in addition to an OTV. The paternal strain is properly genotyped as BB because the OTV does not reduce intensity sufficiently for MouseDivGeno to consider it a VINO. The F1 offspring has an AB genotype and an OTV in heterozygosity but is called AA (Figure 6B). These cases, which we term "cryptic" VINOs, can be detected by analysis of discordant genotypes between parental strains and F1 hybrids. Central OTVs are over-represented in cryptic VINOs, but to a lesser degree than in called VINOs (additional file 7). An edge OTV that is unlikely to result in a VINO call in a homozygous parent may nonetheless provide enough contrast in a heterozygous F1 to result in an incorrect homozygous genotype call. In cases where discrimination between genotype clusters is poor, F1 offspring may be called as N's (Figure 6C).

The concordance rate between predicted and observed genotypes in the F1 hybrid is very high (96.92%) at SNPs genotyped as homozygous for the same allele in both parental strains. The majority of discordant calls are N or V (7315 out of 7373, 99.2%), and only a very small number are due to incorrect heterozygous calls (150, 0.06%) or the wrong homozygous genotype (8, 0.003%). In contrast, the concordance rate is much lower (83.35%) at predicted heterozygous SNPs. Based on the Sanger data, we know that the parental genotypes are correct and thus most of the discordance is due to erroneous genotype calls in the F1 hybrid (Table 3).

### MouseDivGeno can detect VINOs in human genotyping arrays

In order to assess our ability to identify VINOs in other organisms that are routinely genotyped with Affymetrix arrays, we applied MouseDivGeno to 179 Human HapMap 3 samples from the CEU (European), CHB (Chinese), JPT (Japanese) and YRI (African) populations that have both sequence and genotype information available [20]. We further investigated 54 randomly chosen VINOs on chromosome 19 (additional file 8). There were 54 * 179 = 9666 probe/sample combinations, of which 1850 had no genotype called in sequencing data due to a single allele being fixed within a population. In 1619 of these cases, genotypes from the HapMap array calls were substituted.

In the 9435 cases where a HapMap genotype was available, MouseDivGeno calls that had no OTVs were 97.4% concordant (additional file 9). Of the 220 discordant cases, 161 were in areas of below-average sequencing depth, which suggests that off-target SNPs may indeed be present but not detectable. Thus, the discordance rate could be as low as 59/9435 = 0.6%.

When at least one off-target SNP was present, a VINO was called in 17% of cases. In 53.3% of cases, MouseDivGeno instead called the concordant target allele. In another 29.1% of cases, both the target and off-target SNPs were heterozygous and MouseDivGeno called a homozygous allele (i.e. cryptic VINOs). This behavior is expected, as intensity will be reduced only in the probe for the allele carrying the OTV, leading to a large intensity contrast between the alleles that will be interpreted as a homozygous call for the allele not carrying the OTV. For comparison, the discordant rate between HapMap sequencing genotypes and array genotypes is 4% in the 7816 SNP/sample combinations that had a non-N call in both data sets.

In outbred populations such as humans, genotyping of parents would be required to reliably detect heterozygous OTVs. VINOs are enriched in YRI samples, providing better discrimination and counteracting ascertainment bias toward populations of European ancestry (additional files 8, 10).

### VINOs counteract ascertainment bias

The conclusions of genetic research based on genotyping arrays can be influenced by SNP selection (ascertainment) bias. Distances between consecutive SNPs are expected to follow a geometric distribution (additional file 11), with a significant proportion in the 0-12 bp range in species with high levels of variation and large populations size such as the house mouse. Here we demonstrate that this variation can be identified from array intensity data. Variation within genomic DNA targeted by an array probe or a nearby restriction site can alter hybridization intensity sufficiently that we can discriminate the samples harboring previously undetected variation from those that do not. Unlike existing methods that cannot correctly treat these events and may provide an incorrect genotype, MouseDivGeno flags them as VINOs. VINOs are biased in favor of more divergent samples in reverse proportion to the degree to which the genetic variants in a given sample were known and represented on the array at the time of design. Thus VINOs can be used to counteract SNP selection bias.

The Mouse Diversity Array was designed to capture the genetic diversity in the laboratory mouse [1], but the SNP selection was limited to known SNPs and biased due to much deeper knowledge of the variation present in one of the three major subspecies (i.e., *M. m. domesticus*) of *M. musculus*. One method of quantifying this type of ascertainment bias is using SNPs and VINOs with diagnostic alleles that are able to discriminate one subspecies from the other two [4]. There is a significantly larger number of SNPs with diagnostic alleles for *M. m. domesticus *than for the other two subspecies (130526, 63209 and 42598 for *M. m. domesticus, M. m. musculus *and *M. m. castaneus*, respectively). Conversely, VINOs with diagnostic alleles for *M. m. castaneus *are more than triple those for *M. m. domesticus *(16294, 23181 and 56697 for *M. m. domesticus, M. m. musculus *and *M. m. castaneus*, respectively).

**Figure 7:**
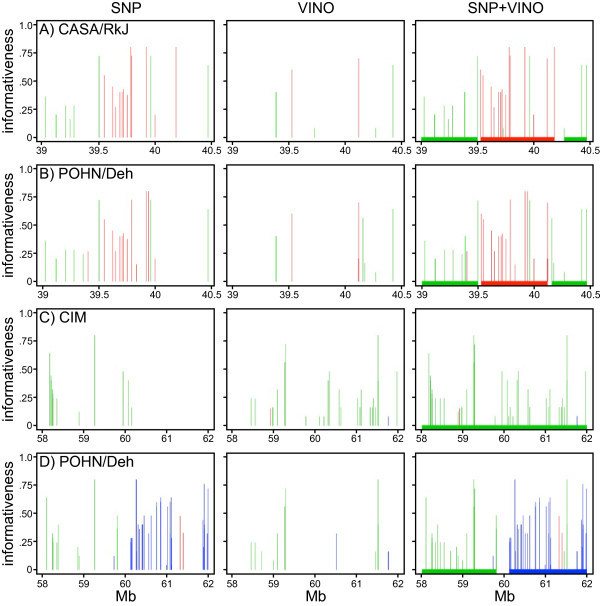
**Use of VINOs improves subspecific origin assignment in mouse**. Diagnostic SNPs and VINOs defined by wild caught mice (first two columns, respectively) and SNPs and VINOs combined (third column) on chromosome 6 (39-40.5 Mb) for strains A) CASA/RkJ and B) POHN/Deh and on chromosome 3 (58-62 Mb) for strains C) CIM and D) POHN/Deh. Blue, red and green vertical lines indicate *M. m. domesticus, M. m. musculus*, and *M. m. castaneus *diagnostic SNPs, respectively. The height of each line represents the score defined by [number of wild animals having diagnostic allele/total animals]. Solid bars along the x-axis in the third column denote the subspecific origin assigned to the interval by a hidden Markov model. In each case, diagnostic VINOs help to refine the boundaries of subspecific origin assignment.

We used SNPs and VINOs with diagnostic alleles to assign a local subspecific origin to each of 162 laboratory strains [4]. For each of these SNPs and VINOs, we determined if a given strain carries the diagnostic allele. Then, using a hidden Markov model, we identified the subspecific origin of each genome segment. Diagnostic SNP information alone might be sufficient to assign the *M. m. domesticus *origin in most regions, however for regions of *M. m. musculus *or *M. m. castaneus *origin diagnostic VINOs enrich the information and help to refine interval boundaries and avoid incorrect assignment (Figure 7).

**Figure 8:**
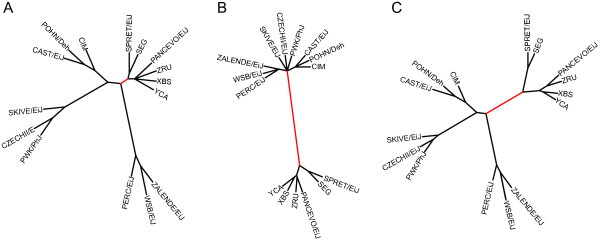
**VINOs improve the topology of phylogenetic trees**. Phylogenetic trees created using A) SNP genotypes only, B) VINOtypes only and C) both SNP genotypes and VINOtypes. The branch highlighted in red separates *M. musculus *and non-*M. musculus *strains and is the most significantly improved by the addition of VINOs.

VINOs are also important for their ability to counteract SNP ascertainment bias when performing phylogenetic studies. This is dramatically illustrated by phylogenetic analysis of several different species of the *Mus *genus (Figure 8). We constructed maximum-likelihood phylogenetic trees using strains derived from *M. musculus, M. spretus, M. spicilegus, M. cypriacus *and *M. macedonicus *(METHODS). When only the standard genotypes are used, the discrimination between non-*M. musculus *species is poor. Furthermore, the length of the *M. m. domesticus *branch is grossly overestimated while non-*M. m. domesticus *branches are underrepresented due to the prevalence of no-calls in these samples (Figure 8A). The opposite result is observed when only VINOtypes are used to construct the tree by converting all genotypes to binary for the presence or absence of a VINO (Figure 8B). When genotypes and VINOtypes are combined, discrimination between taxa increases and a more accurate representation emerges (Figure 8C). These findings are further supported by the approximately 2:1 bias of VINOs in human YRI samples compared the other three HapMap populations (additional file 10). This is consistent with the greater number of genetic variants in African populations that were unknown at the time of the design of the human SNP array.

## Conclusions

We developed the MouseDivGeno package [11] for genotype calling with the Mouse Diversity Array. The genotyping and VINO identification functions can be applied to other hybridization-based genotyping arrays. Genotype calling with MouseDivGeno involves several steps, each of which is reasonable but ad-hoc. The algorithm was derived from a pragmatic process of problem solving, but it is difficult to determine if the approaches used for individual steps and their joint implementation are optimal. In contrast, a modeling based approach, such as that implemented in ALCHEMY software, is appealing because it provides a principled basis for optimization of parameters, and the quality of the genotype calls is encoded in a probability distribution. However, as we have illustrated here, genotype calling with hybridization arrays is an inherently difficult problem that is unlikely to satisfy the strict assumptions of any fully probabilistic model of data generation. There will always be present a subset of loci that fail to conform to modeling expectations but for which visual inspection can provide a reasonable explanation. An advantage of MouseDivGeno lies in the recognition of a frequently occurring anomaly of hybridization intensity (VINOs).

Our ability to identify a VINO is directly correlated with the degree to which OTV(s) decrease hybridization intensity, which depends on a number of factors: the number of OTVs within the genomic target of a probe, the size of the smallest restriction fragment containing the target sequence and whether the target SNP and/or OTV(s) are in heterozygosity. In general OTVs can produce complex intensity patterns with four, five or more distinct clusters (additional file 12) that are highly reproducible but may be difficult to interpret as simple genotype classes because several tightly linked variable loci are involved. However, the reproducible patterns of intensity can be highly informative about the local haplotype that is assayed by the SNP probes. Additional file 12 shows two examples of SNPs for which we have direct sequencing evidence of different haplotypes among OTV-carrying strains (additional file 3). The allelic contrast, although reduced, can still be used to determine the target allele for each VINO sample.

MouseDivGeno also enables downstream analyses that can provide important information on copy-number variation (CNV) and phasing. We have used MouseDivGeno genotypes to determine sample-specific thresholds for CNV detection. We used these thresholds to identify deletions, which are characterized by consecutive VINOs, and have confirmed them using the Sanger data (additional file 13). Tools for haplotype phasing could also be developed for non-inbred samples that rely on probes in which a sample is heterozygous for both the target SNP and the OTV(s).

Our ability to estimate concordance of whole-genome sequencing data with any genotyping software is limited by several factors including coverage depth, reference quality and methods used for assembly and variant identification. We found an enrichment of VINOs within inaccessible regions in the Sanger sequences, however we are unable to determine the validity of these VINOs (without a large-scale direct-sequencing effort). Keane et al. [5] reported a higher proportion of inaccessible regions in wild derived strains and noted that divergence from the mouse reference is a major contributor to inaccessibility. Furthermore, in direct sequencing of BACs from NOD/ShiLtJ, they found 2.8 times more SNPs per base in regions inaccessible to high-throughput sequencing than in accessible regions. They concluded, "at least 30% of all SNPs in the genomes of the strains we sequenced remain to be discovered." Thus, the presence of an OTV or some other hybridization-disrupting feature is likely in these regions, and VINO calls are probably appropriate.

The process of converting continuous data into discrete categories provides the benefit of simplifying information and reducing noise. However, categorization necessarily results in the loss of information and if the underlying model that describes the mapping of genotypes into intensity data is flawed, the resulting genotype calls may be misleading. These considerations suggest that future applications of genotyping array data may benefit from bypassing the genotype-calling step or by augmenting this step and focusing on the direct analysis and interpretation of the hybridization intensity data. For instance, using intensity data for ancestry inference may be advantageous in regions where one haplotype contains OTVs when absent genotype classes may prevent a traditional genotype calling algorithms from differentiating between ancestral strains. In this case, modeling the target intensities as a combination of the intensities of its two ancestral strains would yield more information than inferring ancestry with genotype calls, since there usually is no predefined genotype class for an animal heterozygous between a V or N genotype and another genotype.

We have shown that the methods presented here are generalizable to other Affymetrix arrays in addition to the Mouse Diversity Array. We predict that these methods can be further generalized, with some modification due to differences in chemistry and probe length, to any hybridization array platform. For example, we examined hybridization intensities of common laboratory strains genotyped with the Mouse Universal Genotyping Array [21], a low density Illumina Infinium array with 7851 SNP markers and 50 bp probe sequences. We identified many markers with one or more clusters beyond the standard AA, BB, AB, and N clusters, suggesting OTVs also alter hybridization intensities on the Illumina platform sufficiently for the detection of VINOs (unpublished).

## Methods

### Sample preparation and genotyping

These steps were conducted as previously described [4]. All DNA samples were prepared at the University of North Carolina according to the standard Affymetrix protocol, and all were genotyped using the Mouse Diversity Array [1] at The Jackson Laboratory. CEL files are available at [11].

### Sanger sequencing

We selected 15 SNP probes predicted by visual inspection to have VINOs in multiple strains. For each probe, we selected at least four samples of each genotype (homozygous for allele A, homozygous for B or VINO) for targeted sequencing. Strains for re-sequencing were selected to maximally sample across subspecies and strain type (classical or wild-derived). Primers were designed approximately 200 bp proximal and distal to each probe using PrimerQuest (Integrated DNA Technologies). Probe regions were amplified by PCR and sequenced by automated Sanger sequencing at UNC. Sequences were aligned using SEQUENCHER 4.9 (Gene Codes). Probes, strains and primer sequences used can be found in Table S-four of [4].

### MouseDivGeno

#### Normalization

Normalization adjusts the raw probe intensities to correct for systematic variation within and between arrays that could potentially impact genotype calls. MouseDivGeno offers within-array and quantile normalization features that can be used sequentially or individually. We estimated normalization parameters from the 351 arrays listed in additional file 1. These parameters are specific to the Mouse Diversity Array and are included in the MouseDivGeno software. Application of these normalization steps to other array types will require estimation of these parameters on a set of high quality samples.

Within-array normalization corrects raw intensities for estimated effects of C+G content of the probe and for the lengths of the restriction enzyme fragments generated by genomic sampling [1,22]. We obtained C+G content of each probe from the array design. We obtained expected *NspI *and *StyI *restriction fragment lengths from the mouse genome reference sequence (NCBI mouse genome Build 37). Normalization parameters were obtained by fitting cubic splines [23] with 5 degrees of freedom to the array data.

Quantile normalization is applied to remove variation between arrays [24] and it is justified based on the assumption that the overall distribution of probe intensities should not vary from sample to sample. This assumption may not be valid when taxonomically diverse samples are being studied, in which case we advise against applying this normalization. We pre-computed a reference distribution from good-quality arrays, against which we normalized each array independently. This approach is more computationally efficient than simultaneously normalizing all arrays together [25]. We have included in the MouseDivGeno software a reference distribution derived from the samples in Table S-one of [4].

#### Filtering

Some probes have universally poor performance and cannot be effectively genotyped. Our reference array set includes 211 samples from inbred strains that are most effective for identifying these poor-performing probes. After masking a limited number of strain-specific regions of residual heterozygosity, we excluded probes for which more than 50% of samples have heterozygous genotypes and probes with only one genotype class. In this and related studies [4], we removed these 73525 poorly performing SNPs, leaving a total of 549599 SNPs. We have flagged these poorly performing probe sets and provide an option to keep or remove these SNPs from the genotyping report output of the software.

In addition, we observed that for some probe sets, one strand behaves poorly while the other strand provides good discrimination. We identified 118261 probe sets in which one strand has a silhouette score [26] less than 0.7 and the difference between the silhouette scores across the two strands is greater than 0.2 and excluded intensities of probes on the poorly performing strand from further analyses.

#### Genotype Calling

Genotype calls in MouseDivGeno are based on both contrast and average-intensity data, whereas most existing methods rely solely on the contrast values. MouseDivGeno provides an option to use either the MA or the CCS transformation [3]. CCS transformation shrinks the contrast values in the AA and BB groups and expands the contrast values in AB group as compared to the MA transformation. We used CCS transformation in this paper.

Our genotype-calling algorithm operates in three stages. We initially fit a Gaussian mixture model to the contrast dimension data to find the center of each genotype group and to assign genotypes to those samples having high assignment probability. The initial clustering fits one-, two- and three-component mixture models and determines the number of clusters based on silhouette scores. To initialize the three-component model, we provide the center of the AB group for each SNP obtained from the 351-array reference set. Alternatively the AB center can be set to zero. The AA and BB cluster centers are initially set at the minimum and maximum of the observed contrast intensities. For the two-component model, cluster centers are initialized to the minimum and maximum of the observed contrast intensities. An expectation-maximization (EM) [27] algorithm iteratively fits the center and variance of each group and calculates the probability that each sample belongs to each genotype group. To assign the membership for each sample, we obtain the group-specific threshold. In each genotype group, the threshold is defined by median probability based on samples whose probability of belonging to another group is less than 0.5. Only those sample genotype group probabilities greater than the threshold are assigned at this stage.

After the initial genotype is assigned, we apply a hierarchical clustering method known as 'friends of friends' [28] to assign genotypes to all of the unassigned samples. This step uses both contrast and average-intensity data, and assigns genotypes sequentially. The unassigned sample with the smallest distance to any assigned sample is assigned to the same group as its nearest neighbor. This procedure is repeated until all samples are assigned to genotype classes.

The primary reason for combining the mixture model and hierarchical clustering methods is that each compensates for the other's limitations. The mixture model works best when the variances across genotype groups are balanced. When variances are unbalanced, the algorithm tends to assign samples to the group having the greatest variance. It is typical that the AB group has a larger variance than either of the homozygous groups and this problem is exacerbated when the samples include many inbred strains and few hybrid mice. On the other hand, the nearest neighbor clustering method is most sensitive to "gaps" between distinct groups of sample data points. By combining two methods, we achieve better overall performance than with either method alone.

After assigning genotypes to all samples, we compute the center and variance of each group in two-dimensions (contrast and average intensity), and obtain a confidence score for each sample. We use a Tukey's biweight mean and variance [29] to remove the influence from the outliers. Then the confidence score is calculated as the p-value corresponding to the 2df chi-square distribution of the standardized distance (Mahalonobis distance). Data points that fall below a user defined confidence threshold (in this paper we used 0.005) are then unassigned.

#### VINOtype Calling

To identify low-intensity samples in VINO probes, we compute a VINO score for each sample as described below. If at least one sample exceeds the user-defined VINO threshold at a given SNP, it is assigned a "V" genotype and all other samples are reevaluated at that SNP. We then apply the nearest neighbor algorithm again to search for samples that cluster with the VINO samples and assign a "V" genotype to all of the samples in the new cluster. We initialize this second round of nearest neighbor clustering by removing genotype assignment for samples with average intensity below the overall mean for all samples.

To obtain a VINO score, we need to consider two distances: how far a sample is from the center of its genotype group and how low are the intensities. For each distance we calculate the corresponding p-values and the VINO score is the product of two p-values. The p-value of the first distance is equivalent to the confidence score, and we use (1 - confidence score) so that a high VINO score corresponds to higher likelihood of being a VINO. To calculate the p-value for the second event we compute a z-score for the difference of the average intensity of the sample compared to the mean intensity of its genotype group. The product of these two values is the VINO score for a sample. We use a stringent threshold (score > 0.9999) to assign a sample as a VINO.

At this stage, all sample have been assigned to a genotype group (AA, AB, BB, V or N (no call)). When a sample is assigned to the V genotype class, the confidence score for the genotype call is very low and thus the target SNP should be treated as missing and the VINO treated as a separate variant that is present or absent in each sample.

#### CNV Calling

MouseDivGeno provides two methods to determine CNV: by pairwise comparison of probe set intensities using a Hidden Markov Model, or by providing input files for PENNCNV software [30].

### ALCHEMY and BRLMM-P

We used ALCHEMY version 1.0 [15]. Default parameters were used with the exception of no-call, which was set to 0.04. We used the BRLMM-P 2D algorithm as implemented in the AFFYMETRIX POWER TOOLS software version 1.12.0 [31]. All parameters were set to their default values.

### Sanger Mouse Genomes Project Data

We downloaded Sanger SNP and small indel data sets (2/5/2011 and 7/13/2010 releases, respectively), from the Sanger FTP site. We filtered the Sanger genotype calls, using only those annotated as high confidence (HCG). In a small number of cases, multiple variants affect a single position, in which case we use the most proximal deletion and ignore any other variants overlapped by that deletion. We note that the DNA used by Sanger and our DNA samples are from the same strains but were obtained from different individual mice and that we have previously observed to have low levels of heterogeneity in within-strain comparisons (unpublished).

### Re-annotation of the Mouse Diversity Array

#### Annotation of C57BL/6J

We used BWA [32] to align all probe sequences to the reference genome. BWA was designed as a short-read aligner for high-throughput sequencing data, but works just as well with the Mouse Diversity Array probe sequences, which are between 21 and 25 nucleotides long. We selected BWA over other similar aligners due to its speed, accuracy and support for short insertions and deletions (indels) [33]. If BWA was unable to align any of the probe sequences in a given probe-set with a maximum of one mismatch, that probe-set was annotated as unaligned. For successful alignments, BWA reports full information for a single best alignment and also reports the number of other alignments that are equally good. We combined all information for each probe-set. If there was a single best alignment in at least one strand, we annotated the probe-set with that information. If it was the case that a probe from only one strand was uniquely aligned, or that the best alignment for one strand was different that that of the opposite strand, the probe-set was annotated as such. In all other cases, we annotated the probe-set as non-uniquely aligned.

Next, we identified the proximal and distal *StyI *and *NspI *recognition sites for each uniquely aligned probe-set. We used the OLIGOMATCH program [34] to find all *StyI *and *NspI *recognition sites within the genome and a custom script to identify the proximal and distal sites for each probe-set. It is possible that a change in the reference sequence from build 36 to build 37 has created a new *StyI *or *NspI *recognition site within a probe sequence, thus we provide a separate annotation as to whether this has occurred.

For each uniquely aligned probe-set, we also derived the following annotations: genetic location (in cM) based on the recently updated mouse genetic map [35]; position, type, composition and dbSNP ID of mismatch, if any; ENSEMBL gene, transcript and exon IDs, if the probe-set is located within an exon; C/G content of the probe and the StyI and NspI fragments containing the probe; and number of other zero-mismatch alignments and/or one-mismatch alignments. The full description of the annotation file and all annotations are available for download [11].

#### Annotation of the Sanger strains

We first generated imputed genome sequences of the 14 Sanger strains for which we have Mouse Diversity Array genotypes. We used a custom script to combine SNPs and indels for each strain into a single "patch" file, and a second script to read this patch file and alter the reference genome by changing, inserting or deleting nucleotides at the appropriate locations. We identified regions of low-coverage using criteria defined by the Sanger sequencing project, specifically mapping quality less than 40 (PHRED scale) or coverage depth greater than 150 (indicating duplication) [5].

Next, for each strain we followed the same procedure as was used to annotate the reference genome. We discovered that a total of 1.1% of probe sets for all strains (range 0.8%-2.33%) failed to align due to more than one mismatch or non-uniqueness (additional file 14); these were excluded from further analysis. We maintained reverse maps (from the imputed position in one of the Sanger strains back to the reference position) for determining gene, transcript, exon and SNP IDs. We did not attempt to assign a genetic position to probe-sets in the Sanger strains. We also did not attempt to determine whether polymorphisms led to changes in exon boundaries, and so some probe-sets annotated as being within coding regions may actually be intronic or intergenic, and vice-versa.

### Human genotype data

Human microarray data and SNP calls derived from whole-genome sequences were obtained from the HapMap3 and 1000 Genomes Project FTP site (March 2010 release) [20,36]. We randomly selected 286 samples from the HapMap3, and called genotypes and VINOtypes using MouseDivGeno. We randomly selected for analysis 54 SNPs from chromosome 19 for which there was at least one VINO call.

### Phylogenetic trees of Mus species

#### Samples

*M. m. domesticus*: PERC/EiJ (Peru), WSB/EiJ (Maryland), ZALENDE/EiJ (Switzerland); *M. m. musculus*: CZECHII/EiJ (Czech Republic), PWK/PhJ (Czech Republic), SKIVE/EiJ (wild-derived hybrid between *M. m. domesticus *and *M. m. musculus*, Denmark); *M. m. castaneus*: CAST/EiJ (Thailand), POHN/Deh (Micronesia), CIM (India); *M. spretus*: SPRET/EiJ (Spain), SEG (Spain); *M. spicilegus*: PANCEVO/EiJ (Serbia), ZRU (Ukraine); *M. macedonicus*: XBS (Bulgaria); *M. cypriacus*: YCA (Cyprus).

#### Tree construction

We used the Mouse Phylogeny Browser [37] to select a region of chromosome 11 (37-103 Mb) with minimal introgression from other subspecies in several strains from the three *M. musculus *subspecies. For the SNP-only tree, we converted VINOs to no-calls. For the VINO-only tree, synthetic markers were created at a small distance from each real marker (0.001 cM). Genotypes were converted to C for a VINO call and G otherwise. Both datasets were combined in the SNP + VINO tree. In all cases, genotypes were encoded in NEXUS format. We generated a consensus tree using MRBAYES [38] in an iterative process with the following parameters: 2 substitution types, inverse gamma rate model, MCMC run for a minimum of 100,000 iterations until the standard deviation of split frequencies is below 0.01. Briefly, subsequent runs of MRBAYES were used to more accurately determine branch lengths in the most divergent sub-tree at each stage. This led to the following trees being constructed and manually assembled into the overall tree: non-*M. musculus *samples only, *M. musculus *samples only, *M. m. domesticus *+ *M. m. castaneus *samples only.

## List of Abbreviations

VINO: variable intensity oligonucleotide; SNP: single nucleotide polymorphism; IGP: invariant genomic probe; OTV: off-target variant; RFLP: restriction fragment length polymorphism; CNV: copy number variation.

## Authors' contributions

JPD carried out sequencing, performed intensity analyses and comparison of methods with Sanger and HapMap data and helped to draft the manuscript. HY developed the MouseDivGeno software and carried out subspecific origin assignment and other analyses. KS helped to develop the MouseDivGeno software and carried out pairwise comparisons between methods and analysis of parental and F1 genotype concordance. C-PF and LM developed the VINO visualization tool. FP-MdV and GAC designed the experiments, carried out the analysis on cryptic VINOs and helped to draft the manuscript. All authors read and approved the final manuscript.

## Supplementary Material

Additional file 1 **Description of 351 mouse samples**. Inbred samples used in the VINO analysis are identified in column E. CEL ID corresponds to the name of the Affymetrix CEL file containing raw intensity data, which are available for download [11]. Genetic distance is calculated as the fraction of non-reference allele calls out of all genotype calls. Click here for file

Additional file 2 **Non-homozygous genotype call rates increase with divergence from the reference genome**. A) Genetic distance from the mouse reference genome for 143 laboratory inbred strains (additional file 1). Each strain is shown as a vertical tick mark. Strains are grouped according to their origin are arranged left-to-right in increasing order of genetic distance from the reference. Genetic distance is computed as the fraction of non-reference (non-A allele) genotype calls. B) Genotype calls for each strain. For each strain, the number of SNP probe sets assigned each of the four possible calls (A, B, H or N) are shown as four points of different colors that sum to 526363 SNP probe sets. Click here for file

Additional file 3 **Summary of sequencing of predicted VINOs**. A) Sequencing results for 15 SNPs with samples having predicted OTVs. Forward and reverse strands are shown aligned and the target base is shown in dark black. Each SNP has a different color that corresponds to the mismatches shown in the V1, V2 and V3 columns. B) VINO prediction accuracy. An unrecognized SNP is a probe with an OTV that was not predicted to be a VINO. C) Samples sequenced for each SNP. Colors indicate concordant prediction (red, blue and green), incorrect VINO prediction (yellow) or unrecognized SNP. Click here for file

Additional file 4 **Overall concordance of MouseDivGeno calls with events observed in the Sanger data**. MouseDivGeno Genotypes for 14 Sanger strains classified by the type of event(s) observed in the Sanger data underlying the probe sets. All hybridizations: Both strands affected by the event, or only one strand was affected and the other strand was excluded due to non-alignment; One strand only: Both strands included, but only one strand affected; central OTV: Off-target variant in the center 15-19 bp; edge OTV: Off-target variant in the three bp at either edge of the probe; Inaccessible: SNP falls within an inaccessible region of the Sanger sequence; RFLP 1-1.5K: An RFLP that increases the minimum fragment size to between 1 kb and 1.5 kb; RFLP > 1.5 k: An RFLP that increases the minimum fragment size to greater than 1.5 kb; Cut in Probe: An RFLP that introduces a cut site within the probe sequence. Click here for file

Additional file 5 **Genotype calls by Alchemy and BRLM-P 2D for probes called VINO by MouseDivGeno despite lack of evidence in the Sanger data**. ALCHEMY and BRLMM-P 2D call correct genotypes at a much-reduced rate for the 7073 probe sets for which MouseDivGeno called a VINO with no corresponding evidence in the Sanger data. Click here for file

Additional file 6 **Per-strain concordance of MouseDivGeno calls with events observed in the Sanger data**. MouseDivGeno Genotypes for 14 Sanger strains classified by the type of event(s) observed in the Sanger data underlying the probe sets. Event descriptions are the same as for additional file 4. Click here for file

Additional file 7 **Observed vs. predicted genotype calls in (C57BL/6JxCAST/EiJ)F1, grouped by OTV position**. Genotype calls in (C57BL/6JxCAST/EiJ)F1 are categorized by whether they are concordant (first panel) or discordant (remaining panels), the observed vs. expected genotypes, and the position of the OTV (if any) within the probe set. F1 genotypes are predicted based on CAST/EiJ genotypes, as C57BL/6J is always expected to be AA homozygous. Click here for file

Additional file 8 **MouseDivGeno identifies population-specific VINOs in human samples**. Contrast plots of 9 VINOs identified in HapMap 3 data. Samples in low-intensity clusters are colored by population [20]. Most VINOs are specific to one population or a small number of related populations. Click here for file

Additional file 9 **Concordance between MouseDivGeno calls and 1000 Genomes Project data**. A) Concordance of MouseDivGeno and 1000 Genomes Project sequencing calls for 54 SNPs. B) Breakdown of genotype calls vs. genotypes observed from sequencing data. C) MouseDivGeno VINO calling rate. Click here for file

Additional file 10 **Fraction of VINO calls in each HapMap population**. Each human SNP analyzed in this study is divided into population groups, and the fraction of VINOs called by MouseDivGeno is shown. CEU: Caucasians of European descent from Utah; CHB: Han Chinese from Beijing; JPT: Japanese from Tokyo; YRI: Yoruba in Ibadan, Nigeria. Click here for file

Additional file 11 **The distance between consecutive SNPs follows a geometric distribution**. Histogram of distance between consecutive SNPs in 14 Sanger strains using a bin size of 12 bp. Distances greater than 300 bp are combined in the right-most bin. Click here for file

Additional file 12 **Genotype may be resolved for the target position in some VINOs**. Two examples of SNP probe sets (from the set of VINOs verified by direct sequencing, see additional file 3) for which there are two different low-intensity clusters (red circles) differentiated by the genotype at the target position. A) SNP JAX00258870, for which the low-intensity cluster V1 (RBF/DnJ, TIRANO/EiJ, ZALENDE/EiJ) is homozygous for the G allele at its target SNP, and the low-intensity cluster V2 (BXSB/MpJ and SB/LeJ) is homozygous for the A allele. B) SNP JAX00442587, for which the low-intensity cluster V3 (JF1/Ms, MSM/Ms) is homozygous for the G allele at its target SNP, and the low-intensity cluster V4 (DIK) is homozygous for the A allele. Click here for file

Additional file 13 **VINOs can be used to identify structural variation**. A region of chromosome 12 (approx. 90.847-90.949 Mb) containing a deletion in strain BALB/cJ. Center: sequencing coverage map created from the Sanger data. Each red tick represents a SNP on the Mouse Diversity Array. Top and bottom: contrast plots of intensities for consecutive SNPs. BALB/cJ is highlighted as a red circle, and is located in the low-intensity cluster for the range corresponding to low/no coverage in the Sanger data. Click here for file

Additional file 14 **Summary of unaligned probe sets**. Probe-set sequences were aligned to the imputed genomes for each of 14 Sanger strains using BWA. The fraction of probe non-aligning probe sets is shown. Well-performing probe sets are those included in the present study, while excluded probe sets were removed due to poor performance across the 351 samples in this study. Excluded probe sets are an order of magnitude more likely to be non-aligning. Click here for file
