# Supplementary material for: Discovery of novel variants in genotyping arrays improves genotype retention and reduces ascertainment bias
Source: BMC Genomics. 2012 Jan 19;13:34. doi: 10.1186/1471-2164-13-34 (PMC3305361; doi:10.1186/1471-2164-13-34)
Supplement: Additional file 2 — Non-homozygous genotype call rates increase with divergence from the reference genome. A) Genetic distance from the mouse reference genome for 143 laboratory inbred strains (additional file 1). Each strain is shown as a vertical tick mark. Strains are grouped according to their origin are arranged left-to-right in increasing order of genetic distance from the reference. Genetic distance is computed as the fraction of non-reference (non-A allele) genotype calls. B) Genotype calls for each strain. For each strain, the number of SNP probe sets assigned each of the four possible calls (A, B, H or N) are shown as four points of different colors that sum to 526363 SNP probe sets. [file 1471-2164-13-34-S2.PDF]

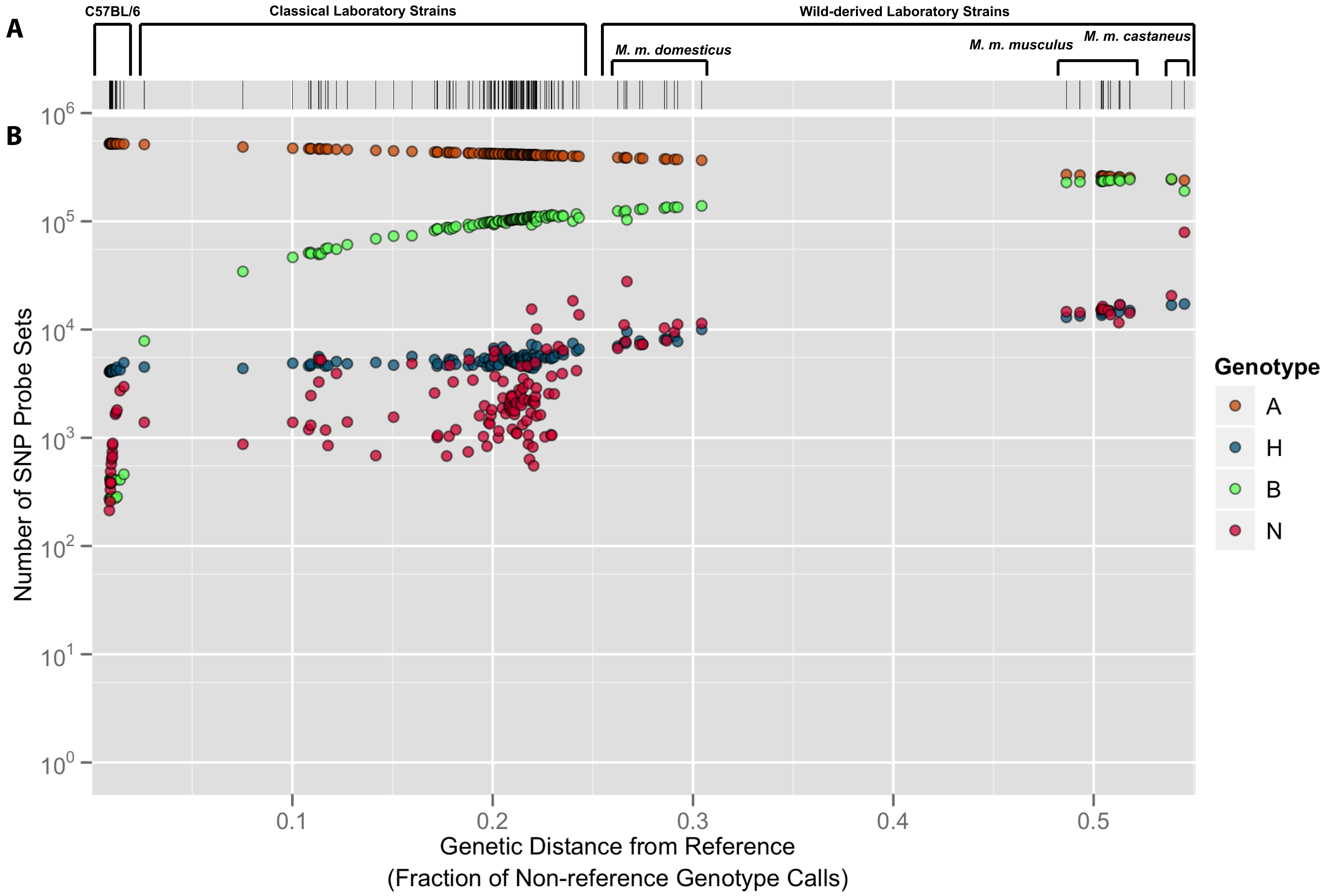

A
B
C57BL/6
Classical Laboratory Strains
Wild-derived Laboratory Strains
M. m. domesticus
M. m. musculus
M. m. castaneus
Number of SNP Probe Sets
10^0
10^1
10^2
10^3
10^4
10^5
10^6
0.1
0.2
0.3
0.4
0.5
Genetic Distance from Reference
(Fraction of Non-reference Genotype Calls)
Genotype
A
H
B
N
