# Supplementary material for: Discovery of novel variants in genotyping arrays improves genotype retention and reduces ascertainment bias
Source: BMC Genomics. 2012 Jan 19;13:34. doi: 10.1186/1471-2164-13-34 (PMC3305361; doi:10.1186/1471-2164-13-34)
Supplement: Additional file 3 — Summary of sequencing of predicted VINOs. A) Sequencing results for 15 SNPs with samples having predicted OTVs. Forward and reverse strands are shown aligned and the target base is shown in dark black. Each SNP has a different color that corresponds to the mismatches shown in the V1, V2 and V3 columns. B) VINO prediction accuracy. An unrecognized SNP is a probe with an OTV that was not predicted to be a VINO. C) Samples sequenced for each SNP. Colors indicate concordant prediction (red, blue and green), incorrect VINO prediction (yellow) or unrecognized SNP. [file 1471-2164-13-34-S3.PDF]

Table S2A. Sequencing results for 15 SNPs with samples having predicted OTVs.

									A						B						V						V1			V2			V3		
JAX SNP ID	Chr	Sequence (F/R)	Total Samples	Failed	Good	True-Positive	Unrecognized SNP	VINO Miscall	Predicted	Failed	Actual	True-Positive	Unrecognized SNP	Miscall	Predicted	Failed	Actual	True-Positive	Unrecognized SNP	Miscall	Predicted	Failed	Actual	True-Positive	Miscall	Unrecognized SNP	True-Positive	Unrecognized SNP	Genotype	True-Positive	Unrecognized SNP	Genotype	True-Positive	Unrecognized SNP	Genotype
		1 5 10 15 20 25 30																																	
**JAX00241414**	1	ACAAAGGTACCAAAGA**C**TAT**C**AGAT<br>GTACCAAAGA**C**TAT**C**AGATAACCAT	23	0	23	23	0	0	4	0	4	4	0	0	8	0	8	8	0	0	11	0	11	11	0	0	11	0	**17C>G, 21C>T**						
**JAX00258870**	1	AGAGGTTGTTT**C**GAAC**A**AA**G**TCCAG<br>GTTGTTT**C**GAAC**A**AA**G**TCCAGAGA**A**	24	1	23	20	3	0	12	0	12	12	0	0	7	1	3	3	3	0	5	0	8	5	0	3	3	3	**17A>G, 28A>C**	3	0	**17A>G, 20G>**	2	0	**12C>T**
**JAX00518420**	3	TTCAGGTCC**A**TT**T**CTAAGTCAAGAG<br>GGTCC**A**TT**T**CTAAGTCAAGAGGCAT	20	0	20	20	0	0	8	0	8	8	0	0	8	0	8	8	0	0	4	0	4	4	0	0	4	0	**10A>T**, **13T>C**						
**JAX00567838**	4	AGGTAC**G**TCGTCCT**G**ACCACAAAGC<br>GTAC**G**TCGTCCT**G**ACCACAAAGCAT	21	0	21	21	0	0	12	0	12	12	0	0	4	0	4	4	0	0	5	0	5	5	0	0	5	0	**7G>A**, **15G>A**						
**JAX00149491**	7	CCTCTTTT**TG**AGTTCATCTTCAACC<br>CGAAC**C**CCCCTCTTTT**TG**AGTTCAT	17	3	14	10	0	4	5	1	4	4	0	0	5	1	8	4	0	4	7	1	2	2	4	0	2	0	**6C>A, 18G>T**						
**JAX00156936**	7	CTTCCAGTAAAATA**T**GC**G**GTGTACA<br>TCCAGTAAAATA**T**GC**G**GTGTACACC	21	0	21	21	0	0	5	0	5	5	0	0	9	0	9	9	0	0	7	0	7	7	0	0	7	0	**15T>C, 18G>A**						
**JAX00642397**	7	CTGGTATC**G**TGATTAT**C**ATTTTTGG<br>CTGGTATC**G**TGATTAT**C**ATTTTTGG	23	2	21	21	0	0	12	0	12	12	0	0	6	0	6	6	0	0	5	2	3	3	0	0	3	0	**9G>A**						
**JAX00649359**	7	TGGGTATGG**CA**CCTTTTAACTACAC<br>GGTATGG**CA**CCTTTTAACTACACAG	21	2	19	19	0	0	10	0	10	10	0	0	7	1	6	6	0	0	4	1	3	3	0	0	3	0	**10C>T**, **11A>G**						
**JAX00300473**	10	TTGTAAA**TA**CATAGTTGCAAATCCA<br>CAGATTGTAAA**TA**CATAGTTGCAAA	20	1	19	19	0	0	7	0	7	7	0	0	9	0	9	9	0	0	4	1	3	3	0	0	3	0	**12T>C**, **13A>C**						
**JAX00031437**	11	CAT**C**TC**G**AAGTC**A**TCCTCACAGAGG<br>CAT**C**TC**G**AAGTC**A**TCCTCACAGAGG	21	1	20	20	0	0	5	0	5	5	0	0	6	1	5	5	0	0	10	0	10	10	0	0	7	0	**7G>A**	3	0	**4C>T**, **7G>A**			
**JAX00303026**	11	CATGGCCTTCCGAACC**G**TCTCTTCG<br>TCCGAACC**G**TCTCTTCGTCTCCCAG	22	0	22	30	0	0	10	0	10	10	0	0	4	0	4	4	0	0	8	0	8	8	0	0	8	0	RFLP						
**JAX00376696**	14	ACCAG**C**GA**G**CTCCT**G**TTTAAGGT**A**A<br>ACCAG**C**GA**G**CTCCT**G**TTTAAGGT**A**A	19	0	19	14	5	0	11	0	6	6	5	0	4	0	3	3	0	0	5	0	10	5	0	5	5	5	**24A>G**	3	0	**6C>T**	2	0	**6C>T**, **15G>A**
**JAX00434064**	17	CAACAACAGTCC**T**GATTCCA**A**AATC<br>AACAGTCC**T**GATTCCA**A**AATCTACA	23	2	21	21	0	0	13	1	12	12	0	0	4	0	4	4	0	0	6	1	5	5	0	0	5	0	**13T>C, 21A>G**						
**JAX00442587**	17	TTATTGGCTTTCA**CA**AT**C**GGAGTTT<br>ATTGGCTTTCA**CA**AT**C**GGAGTTTCA	21	2	19	19	0	0	12	0	12	12	0	0	4	0	4	4	0	0	6	3	3	3	0	0	2	0	**14C>T**, **15A>G**	1	0	**18C>T**			
**JAX00470882**	19	TG**GT**C**A**AA**C**ATCTTTCGAGGGTCAG<br>TG**GT**C**A**AA**C**ATCTTTCGAGGGTCAG	22	1	21	20	1	0	12	0	11	11	1	0	4	0	4	4	0	0	6	1	6	5	0	1	5	0	**3G>C**, **4T>A**, **6A>G**, **9C>A**	1	1	**3G>C**, **4T>A**			
**Totals**			318	15	303	298	9	4	138	2	130	130	6	0	89	4	85	81	3	4	93	10	88	79	4	9									

Table S2B. VINO prediction accuracy

Prediction	Correct Call	Incorrect Call	Unrecognized SNP	Error Rate
**No OTV**	211	0	9	4.1%
**OTV**	79	4	0	4.8%

Table S2C. Samples sequenced for each SNP.

JAX00241414	JAX00258870	JAX00518420	JAX00567838	JAX00149491	JAX00156936	JAX00642397	JAX00649359	JAX00300473	JAX00031437	JAX00303026	JAX00376696	JAX00434064	JAX00442587	JAX00470882
WSB/EiJ	129S1SvImJ	129S1SvImJ	129S1SvImJ	129S1SvImJ	A/J	129S1SvImJ	129S1SvImJ	A/J	129S1SvImJ	129S1SvImJ	129S1SvImJ	A/J	129S1SvImJ	129S1SvImJ
PERA/EiJ	A/J	A/J	A/J	C57BL/6J	C57BL/6J	A/J	A/J	C57BL/6J	C57BL/6J	A/J	A/J	C57BL/6J	A/J	C57BL/6J
C57BL/6J	C57BL/6J	C57BL/6J	C57BL/6J	NZO/HILtJ	NZO/HILtJ	C57BL/6J	C57BL/6J	NOD/LtJ	NOD/LtJ	C57BL/6J	C57BL/6J	NOD/LtJ	C57BL/6J	NZO/HILtJ
CALB/RkJ	NOD/LtJ	NOD/LtJ	NOD/LtJ	WSB/EiJ	SF/CAMEiJ	NOD/LtJ	NOD/LtJ	NZO/HILtJ	LEWES/EiJ	NOD/LtJ	NOD/LtJ	NZO/HILtJ	NOD/LtJ	WSB/EiJ
CAST/EiJ	NZO/HILtJ	NZO/HILtJ	NZO/HILtJ	A/J	MOR/RkJ	NZO/HILtJ	NZO/HILtJ	LEWES/EiJ	MOLD/RkJ	NZO/HILtJ	NZO/HILtJ	WSB/EiJ	NZO/HILtJ	PWK/PhJ
TIRANO/EiJ	WSB/EiJ	WSB/EiJ	SF/CAMEiJ	NOD/LtJ	129S1SvImJ	WSB/EiJ	WSB/EiJ	MOLD/RkJ	A/J	WSB/EiJ	WSB/EiJ	PWK/PhJ	WSB/EiJ	JF1/Ms
A/J	PWK/PhJ	CAST/EiJ	PWK/PhJ	LEWES/EiJ	NOD/LtJ	CAST/EiJ	MOLD/RkJ	ALR/LtJ	NZO/HILtJ	PWK/PhJ	PWK/PhJ	JF1/Ms	CZECHI/EiJ	CAST/EiJ
AKR/J	JF1/Ms	POHN/Deh	JF1/Ms	PWK/PhJ	WSB/EiJ	STUP	CASA/RkJ	RIIIS/J	WSB/EiJ	MOLF/EiJ	CAST/EiJ	BULS	CAST/EiJ	MSM/Ms
TIRANO/EiJ	CKN	AKR/J	CAST/EiJ	CIM	CZECHI/EiJ	POHN/Deh	BUSNA	WSB/EiJ	CAST/EiJ	CAST/EiJ	P/J	MSM/Ms	BULS	BULS
BIK	BULS	PERC/EiJ	SKIVE/EiJ	CKS	JF1/Ms	SKIVE/EiJ	TIRANO/EiJ	PWK/PhJ	TIRANO/EiJ	TIRANO/EiJ	SOD1/EiJ	SKIVE/EiJ	POHN/Deh	SKIVE/EiJ
DIK	SKIVE/EiJ	PWK/PhJ	MSM/Ms	JF1/Ms	CAST/EiJ	STUF	NH/KiPtJ	JF1/Ms	PWK/PhJ	129X1/SvJ	PERC/EiJ	STUF	STUF	STUF
POHN/Deh	POHN/Deh	JF1/Ms	POHN/Deh	BUSNA	SKIVE/EiJ	TIRANO/EiJ	PWK/PhJ	BULS	JF1/Ms	BALB/cByJ	MGA	TIRANO/EiJ	TIRANO/EiJ	ALR/LtJ
MOLD/RkJ	BUB/BnJ	SKIVE/EiJ	I/LnJ	BULS	STUF	PWK/PhJ	JF1/Ms	MSM/Ms	MDG	BALB/cJ	RBA/DnJ	129S1SvImJ	PWK/PhJ	ALS/LtJ
CZECHI/EiJ	C3H/HeJ	MSM/Ms	ISS	MOLD/RkJ	TIRANO/EiJ	JF1/Ms	SKIVE/EiJ	SKIVE/EiJ	CZECHI/EiJ	I/LnJ	BULS	129P1/ReJ	MOLD/RkJ	BUB/BnJ
JF1/Ms	SF/CAMEiJ	STUF	WSB/EiJ	MGA	PWK/PhJ	PWD/PhJ	MSM/Ms	STUF	CZECHII/EiJ	YBR/EiJ	CZECHII/EiJ	LEWES/EiJ	STUP	C3H/HeJ
PWK/PhJ	BXSB/MpJ	TIRANO/EiJ	PERA/EiJ	CAST/EiJ	MOLD/RkJ	MOLD/RkJ	BULS	CAST/EiJ	PWD/PhJ	DDK	JF1/Ms	PERA/EiJ	SKIVE/EiJ	A/J
STUP	RBF/DnJ	CZECHII/EiJ	RBF/DnJ	SKIVE/EiJ	PWD/PhJ	BULS	CAST/EiJ	CASA/RkJ	BULS	CZECHI/EiJ	MSM/Ms	BPN/3J	JF1/Ms	NOD/LtJ
SKIVE/EiJ	SB/LeJ	BUSNA	LEWES/EiJ		MGA	MSM/Ms	STUP	CALB/RkJ	MSM/Ms	JF1/Ms	SKIVE/EiJ	P/J	MSM/Ms	A/HeJ
MGA	TIRANO/EiJ	BULS	RBA/DnJ		BUSNA	PL/J	STUS	CKS	SKIVE/EiJ	CZECHII/EiJ	STUF	CAST/EiJ	DIK	RBB/DnJ
MSM/Ms	ZALENDE/EiJ	STUS	TIRANO/EiJ		BULS	PERC/EiJ	CKS		STUF	BULS		CASA/RkJ	BIK/g	DCP
SKIVE/EiJ	MOLD/RkJ		ZALENDE/EiJ		MSM/Ms	RBB/DnJ	(PWKxCAST)F1 m005		MBS	MSM/Ms		CALB/RkJ	MGA	TIRANO/EiJ
STUF	STUS					CTP				SKIVE/EiJ		CKS		DGA
BULS	MSM/Ms					SM/J						BDP/J		
	CAST/EiJ													

Reference Allele
Variant Allele
True Positive VINO
False Positive VINO
Unrecognized SNP
Failed
