# Supplementary material for: Discovery of novel variants in genotyping arrays improves genotype retention and reduces ascertainment bias
Source: BMC Genomics. 2012 Jan 19;13:34. doi: 10.1186/1471-2164-13-34 (PMC3305361; doi:10.1186/1471-2164-13-34)
Supplement: Additional file 4 — Overall concordance of MouseDivGeno calls with events observed in the Sanger data. MouseDivGeno Genotypes for 14 Sanger strains classified by the type of event(s) observed in the Sanger data underlying the probe sets. All hybridizations: Both strands affected by the event, or only one strand was affected and the other strand was excluded due to non-alignment; One strand only: Both strands included, but only one strand affected; central OTV: Off-target variant in the center 15-19 bp; edge OTV: Off-target variant in the three bp at either edge of the probe; Inaccessible: SNP falls within an inaccessible region of the Sanger sequence; RFLP 1-1.5K: An RFLP that increases the minimum fragment size to between 1 kb and 1.5 kb; RFLP > 1.5 k: An RFLP that increases the minimum fragment size to greater than 1.5 kb; Cut in Probe: An RFLP that introduces a cut site within the probe sequence. [file 1471-2164-13-34-S4.PDF]

Table S3. Overall concordance of MouseDivGeno calls with events observed in the Sanger data.

		MouseDivGeno					
**Sanger**		**Homozygous, Concordant**	**Homozygous, Discordant**	**Heterozygous**	**V**	**N**	**Total**
**Events Affecting All Hybridizations**							
**Both Strands, Single Event, Same Types**							
Central OTV		15,785	962	2,344	12,158	4,148	35,397
Edge OTV		10,985	57	298	197	586	12,123
Inaccessible		485	242	456	2,252	8,283	11,718
RFLP 1-1.5K		52,754	321	2,218	2,196	4,052	61,541
RFLP > 1.5K		24,977	670	2,162	3,440	2,714	33,963
Cut in Probe		270	7	2	2	4	285
	**Total**	105,256	2,259	7,480	20,245	19,787	155,027
**One Strand, Single Event**							
Central OTV		1,032	413	186	838	340	2,809
Edge OTV		752	243	52	101	138	1,286
Other		109	34	13	21	21	198
	**Total**	1,893	690	251	960	499	4,293
**Multiple Events**		1,729	198	323	985	475	3,710
**Both Strands, Single Event, Different Types**							
Central OTV/Edge OTV		7,393	84	417	913	1,016	9,823
Central OTV/Multiple		227	12	30	121	73	463
Edge OTV/Multiple		134	1	7	11	15	168
RFLP 1-1.5K/Multiple		1,149	17	74	117	159	1,516
RFLP > 1.5K/Multiple		522	30	79	152	109	892
Other		617	6	35	45	125	828
	**Total**	10,042	150	642	1,359	1,497	13,690
		118920	3297	8696	23549	22258	176720
**Events Affecting One Strand Only**							
Central OTV		7,568	66	349	251	660	8,894
Edge OTV		35,954	78	859	215	1,332	38,438
Inaccessible		161	61	13	18	11	264
RFLP 1-1.5K		20,539	21	263	38	242	21,103
RFLP > 1.5K		53,362	94	704	89	706	54,955
Cut in Probe		2,523	7	41	2	29	2,602
Multiple		215	6	9	8	20	258
	**Total**	120,322	333	2,238	621	3,000	126,514
**No Events**		6,877,070	8,941	80,054	7,073	75,711	7,048,849
**Excluded Probe Sets**							
Both Strans Unaligned		0	0	737	3,600	5,390	9,727
Non-unique Alignment		0	0	579	761	5,932	7,272
	**Total**	0	0	1,316	4,361	11,322	16,999
**Table Subtotals**		**7,116,312**	**12,571**	**92,304**	**35,604**	**112,291**	**7,369,082**
