# Supplementary material for: Discovery of novel variants in genotyping arrays improves genotype retention and reduces ascertainment bias
Source: BMC Genomics. 2012 Jan 19;13:34. doi: 10.1186/1471-2164-13-34 (PMC3305361; doi:10.1186/1471-2164-13-34)
Supplement: Additional file 5 — Genotype calls by Alchemy and BRLM-P 2D for probes called VINO by MouseDivGeno despite lack of evidence in the Sanger data. ALCHEMY and BRLMM-P 2D call correct genotypes at a much-reduced rate for the 7073 probe sets for which MouseDivGeno called a VINO with no corresponding evidence in the Sanger data. [file 1471-2164-13-34-S5.PDF]

Table S4. Genotype calls by Alchemy and BRLM-P 2D for probes called VINO by MouseDivGeno despite lack of evidence in Sanger data.

Genotype Call	Alchemy		BRLMM-P 2D	
**AA or BB, Concordant**	2,919	41.3%	2,183	30.9%
**AA or BB, Discordant**	67	0.9%	50	0.7%
**AB**	658	9.3%	2,100	29.7%
**N**	3,429	48.5%	2,740	38.7%
