# Supplementary material for: Discovery of novel variants in genotyping arrays improves genotype retention and reduces ascertainment bias
Source: BMC Genomics. 2012 Jan 19;13:34. doi: 10.1186/1471-2164-13-34 (PMC3305361; doi:10.1186/1471-2164-13-34)
Supplement: Additional file 7 — Observed vs. predicted genotype calls in (C57BL/6JxCAST/EiJ)F1, grouped by OTV position. Genotype calls in (C57BL/6JxCAST/EiJ)F1 are categorized by whether they are concordant (first panel) or discordant (remaining panels), the observed vs. expected genotypes, and the position of the OTV (if any) within the probe set. F1 genotypes are predicted based on CAST/EiJ genotypes, as C57BL/6J is always expected to be AA homozygous. [file 1471-2164-13-34-S7.PDF]

Table S6. Observed vs predicted genotype calls in (C57BL/6JxCAST/EiJ)F1 by OTV position.

F1 Genotype Calls

	Concordant		Expected AA			Expected AB			CAST = AB				CAST = V					
**OTV Position**	**AA**	**AB**	**BB**	**AB**	**V**	**AA**	**BB**	**V**	**AA**	**BB**	**AB**	**V**	**AA**	**BB**	**AB**	**V**	**N**	**Margins**
**No OTV**	227,016	151,501	1	29	66	5,532	8,400	161	4,575	4	5,613	36	5,865	16	1,510	441	61,726	472,492
**0**	1,774	1,356	0	1		138	77	5	61	0	66	1	116	2	13	10	603	4,223
**1**	2,817	2,100	0	0	0	261	125	3	117	0	90	3	236	0	37	16	1,107	6,912
**2**	1,531	1,047	0	0	1	259	48	2	119	0	44	1	309	0	21	30	890	4,302
**3**	778	504	0	0	0	283	21	6	99	0	30	3	488	0	22	42	723	2,999
**4**	408	223	0	0	2	255	8	2	115	0	15	1	600	0	5	44	616	2,294
**5**	232	85	0	0	0	215	10	5	118	0	12	1	616	0	8	60	501	1,863
**6**	158	86	0	0	0	205	4	4	76	0	8	1	669	0	3	61	460	1,735
**7**	153	84	0	0	0	218	2	2	110	0	10	3	785	0	4	77	483	1,931
**8**	117	61	0	0	1	181	1	0	75	0	1	0	580	0	5	73	400	1,495
**9**	97	35	0	1	1	199	2	0	67	0	8	2	543	0	4	64	369	1,392
**10**	94	50	0	0	1	171	1	2	70	0	3	1	494	0	3	60	342	1,292
**11**	76	25	0	0	0	129	3	1	44	0	1	2	345	0	2	31	223	882
**12**	34	17	0	0	0	35	0	0	14	0	1	0	153	0	3	17	86	360
	235,285	157,174	1	31	72	8,081	8,702	193	5,660	4	5,902	55	11,799	18	1,640	1,026	68,529	504,172
																	**Excluded**	22,191
																		526,363
