# Supplementary figures and images for: Discovery of novel variants in genotyping arrays improves genotype retention and reduces ascertainment bias
Source: BMC Genomics. 2012 Jan 19;13:34. doi: 10.1186/1471-2164-13-34 (PMC3305361; doi:10.1186/1471-2164-13-34)

○ AA ○ AB ○ BB ● VINO  
● CEU ● CHD ● GIH ● MEX ● YRI ● CHB ● JPT ● MKK ● ASW ● TSI ● LWK

SNP\_A-2167587

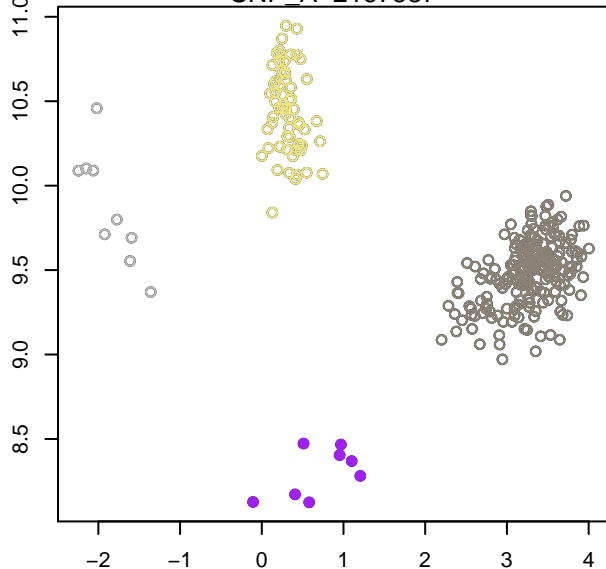

SNP\_A-2176547

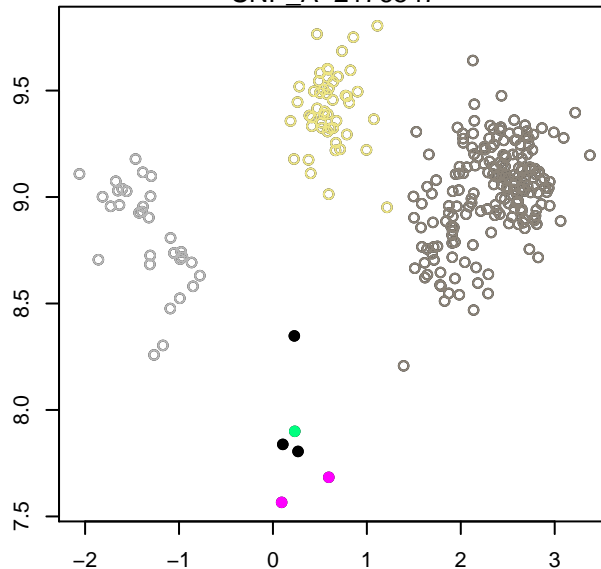

SNP\_A-2262511

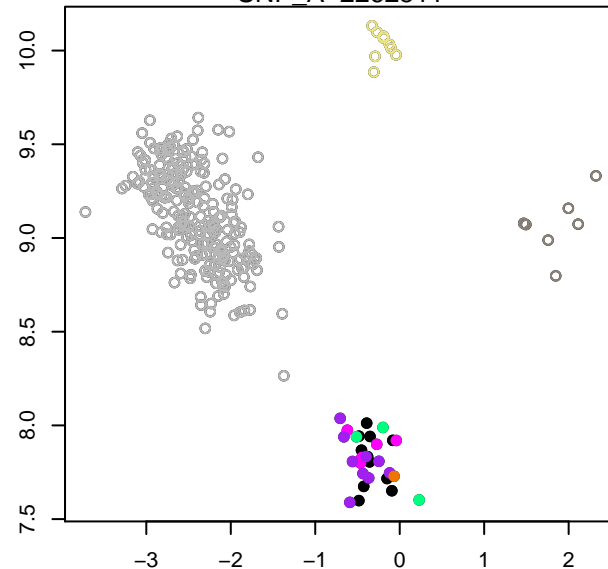

SNP\_A-8534276

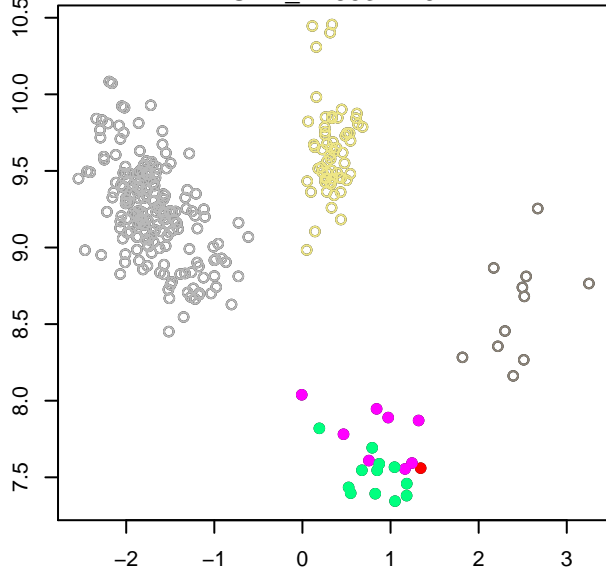

SNP\_A-8302015

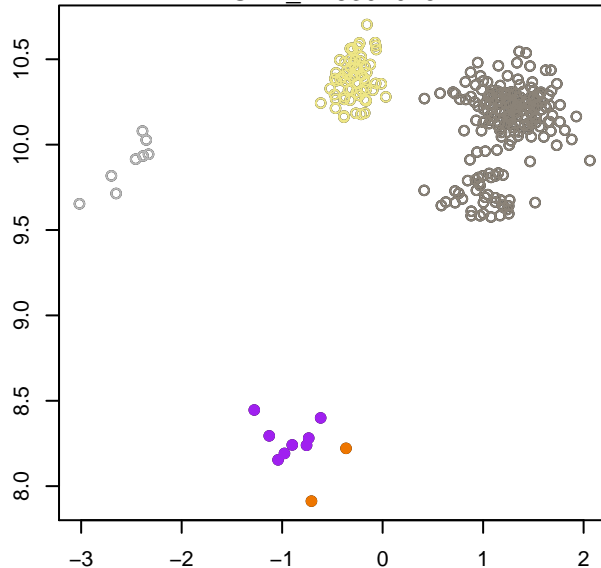

SNP\_A-8310429

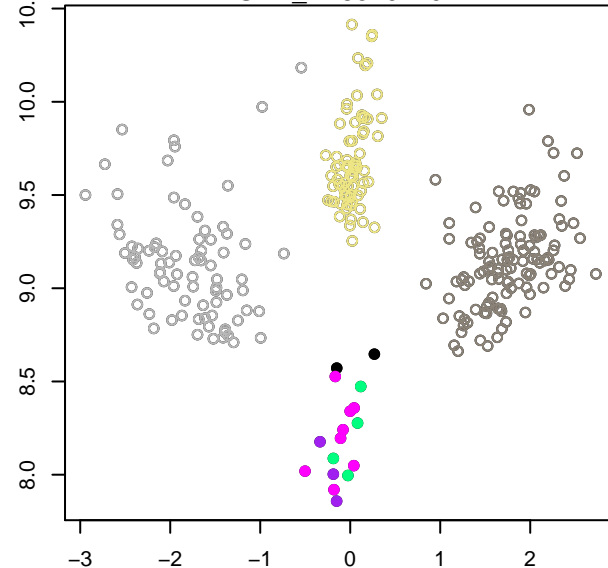

SNP\_A-8405350

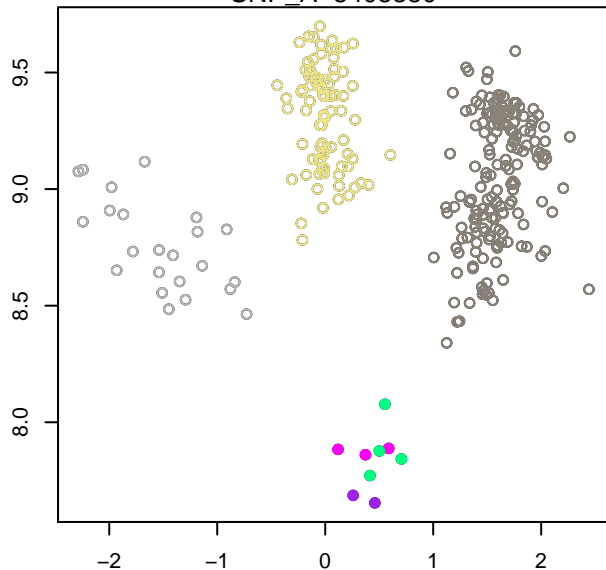

SNP\_A-8418784

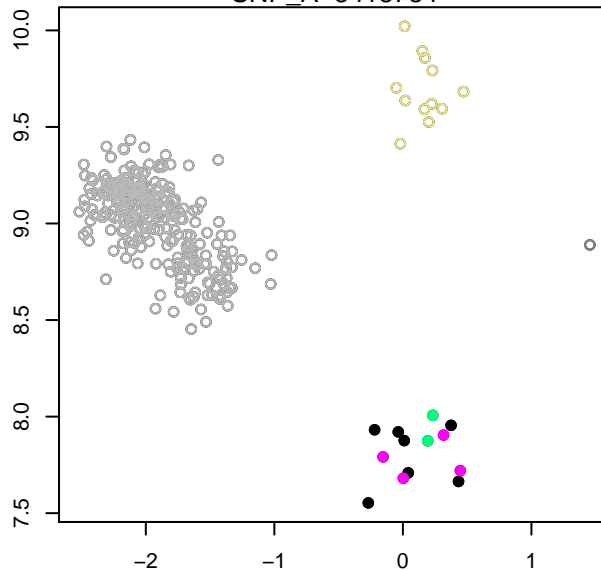

SNP\_A-8515850

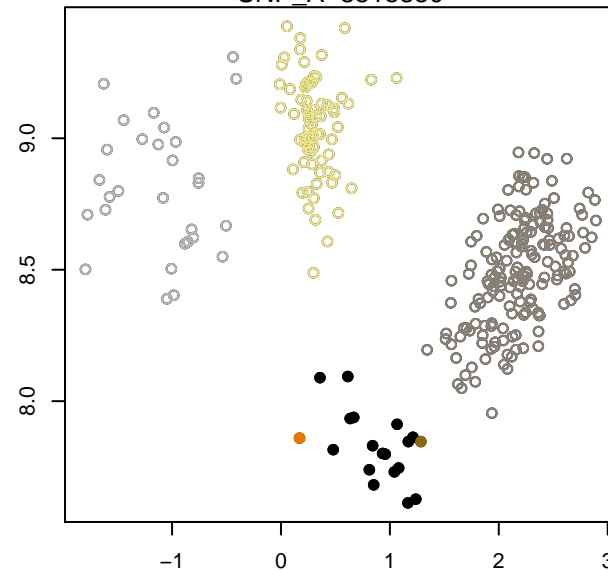

Supplement: Additional file 8 — MouseDivGeno identifies population-specific VINOs in human samples. Contrast plots of 9 VINOs identified in HapMap 3 data. Samples in low-intensity clusters are colored by population [20]. Most VINOs are specific to one population or a small number of related populations. [file 1471-2164-13-34-S8.PDF]

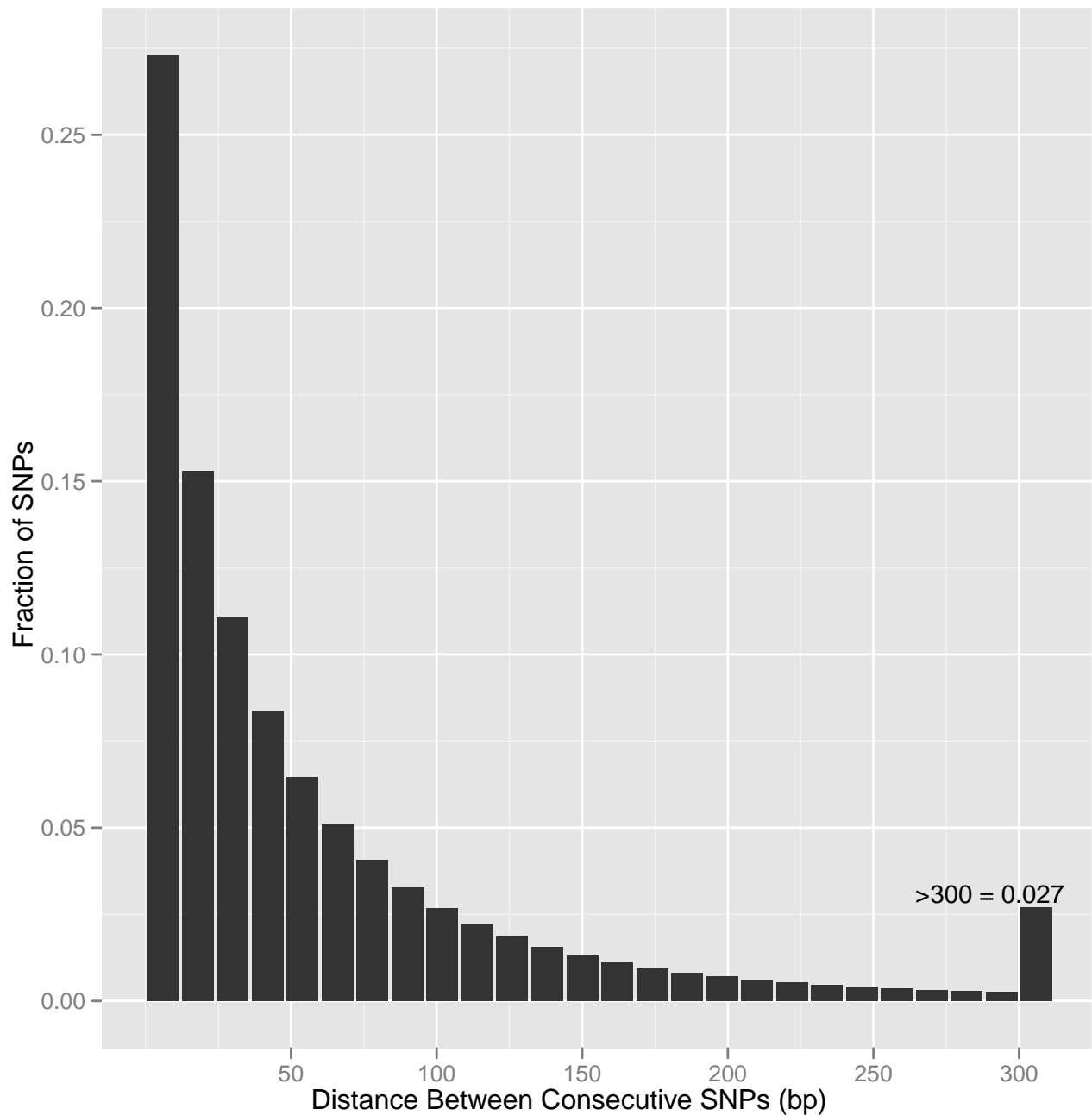

Supplement: Additional file 10 — Fraction of VINO calls in each HapMap population. Each human SNP analyzed in this study is divided into population groups, and the fraction of VINOs called by MouseDivGeno is shown. CEU: Caucasians of European descent from Utah; CHB: Han Chinese from Beijing; JPT: Japanese from Tokyo; YRI: Yoruba in Ibadan, Nigeria. [file 1471-2164-13-34-S10.PDF]

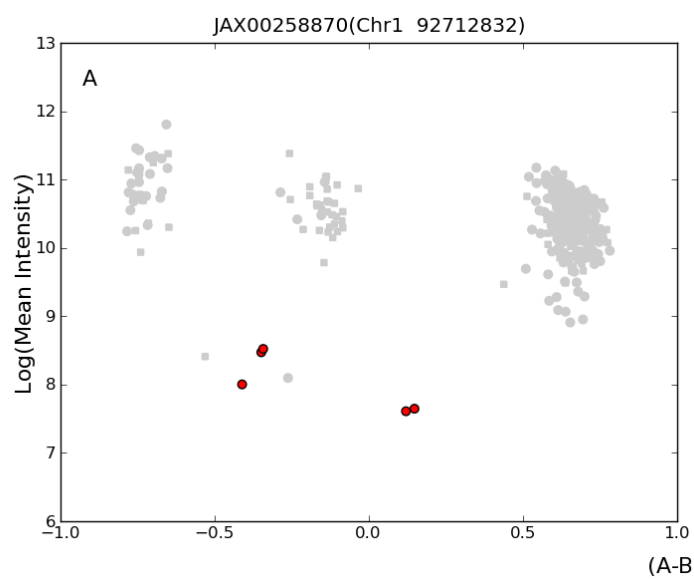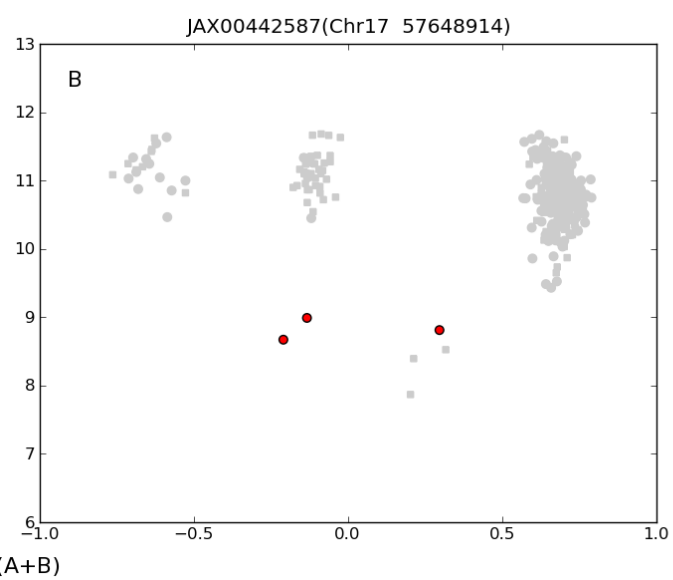

Supplement: Additional file 12 — Genotype may be resolved for the target position in some VINOs. Two examples of SNP probe sets (from the set of VINOs verified by direct sequencing, see additional file 3) for which there are two different low-intensity clusters (red circles) differentiated by the genotype at the target position. A) SNP JAX00258870, for which the low-intensity cluster V1 (RBF/DnJ, TIRANO/EiJ, ZALENDE/EiJ) is homozygous for the G allele at its target SNP, and the low-intensity cluster V2 (BXSB/MpJ and SB/LeJ) is homozygous for the A allele. B) SNP JAX00442587, for which the low-intensity cluster V3 (JF1/Ms, MSM/Ms) is homozygous for the G allele at its target SNP, and the low-intensity cluster V4 (DIK) is homozygous for the A allele. [file 1471-2164-13-34-S12.PDF]

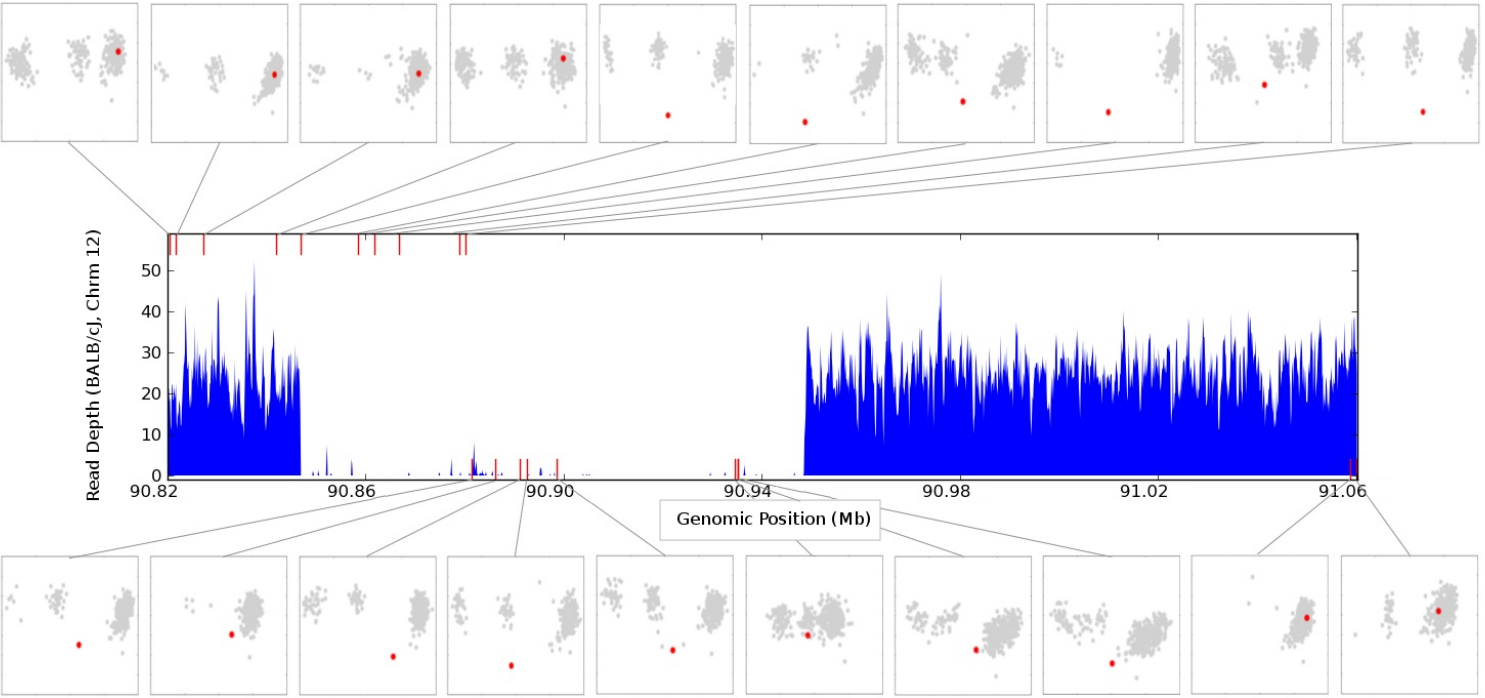

Supplement: Additional file 13 — VINOs can be used to identify structural variation. A region of chromosome 12 (approx. 90.847-90.949 Mb) containing a deletion in strain BALB/cJ. Center: sequencing coverage map created from the Sanger data. Each red tick represents a SNP on the Mouse Diversity Array. Top and bottom: contrast plots of intensities for consecutive SNPs. BALB/cJ is highlighted as a red circle, and is located in the low-intensity cluster for the range corresponding to low/no coverage in the Sanger data. [file 1471-2164-13-34-S13.PDF]
