# Supplementary material for: Discovery of novel variants in genotyping arrays improves genotype retention and reduces ascertainment bias
Source: BMC Genomics. 2012 Jan 19;13:34. doi: 10.1186/1471-2164-13-34 (PMC3305361; doi:10.1186/1471-2164-13-34)
Supplement: Additional file 9 — Concordance between MouseDivGeno calls and 1000 Genomes Project data. A) Concordance of MouseDivGeno and 1000 Genomes Project sequencing calls for 54 SNPs. B) Breakdown of genotype calls vs. genotypes observed from sequencing data. C) MouseDivGeno VINO calling rate. [file 1471-2164-13-34-S9.PDF]

Table S7A. Concordance of MouseDivGeno and 1000 Genomes Project sequencing calls for 54 SNPs.

	1000 Genomes				
**MouseDivGeno**	**AA**	**BB**	**AB**	**Low Coverage**	**Total**
**AA**	2,800	2	187	65	3,054
**BB**	150	3,540	105	40	3,835
**AB**	49	47	2,037	28	2,161
**V**	74	91	10	3	178
**Total**	3,073	3,680	2,339	136	9,228

Table S7B. Details of discordant calls.

	SNPs	%	Cases	%
**Total**	54		9435	
**Concordant, No Off-target SNPs**	54	100.0%	8266	**87.6%**
**Discordant, No Off-target SNPs**	44	81.5%	220	**2.3%**
**Coverage between 0 and 1 SD less than mean**			97	1.0%
**Coverage more than 1 SD less than mean**			64	0.7%
**Coverage >= mean**			59	0.6%
**CalledABH, observed AA or BB**			21	
**Called AA or BB, observed AB**			27	
**Called V, observed AA or BB**			6	
**Called V, observed AB**			4	
**Called AA or BB, observed opposite allele**			1	
**Concordant, Off-target SNPs**	35	64.8%	161	**1.7%**
**Discordant, Off-target SNPs**	36	66.7%	788	**8.4%**
**Concordant target genotype call**			506	5.4%
**Called AA or BB, observed opposite allele and off-target Het SNP**			276	2.9%
**Other**			6	0.1%

Table S7C. MouseDivGeno VINO calling rate.

MouseDivGeno Call	CEU	CHB/JPT	YRI
**Concordant VINO Calls**	31	53	82
**Off-Target SNPs**	202	289	482
**VINO Call Rate**	15.3%	18.3%	17.0%
