# Supplementary material for: Discovery of novel variants in genotyping arrays improves genotype retention and reduces ascertainment bias
Source: BMC Genomics. 2012 Jan 19;13:34. doi: 10.1186/1471-2164-13-34 (PMC3305361; doi:10.1186/1471-2164-13-34)
Supplement: Additional file 11 — The distance between consecutive SNPs follows a geometric distribution. Histogram of distance between consecutive SNPs in 14 Sanger strains using a bin size of 12 bp. Distances greater than 300 bp are combined in the right-most bin. [file 1471-2164-13-34-S11.PDF]

Table S8. Fraction of VINO calls in each HapMap population.

SNP ID	CEU	CHB/JPT	YRI
SNP_A-1939610			3.40%
SNP_A-4260229			1.70%
SNP_A-4260524	3.30%		
SNP_A-4260951			1.80%
SNP_A-1961751	3.30%		
SNP_A-2115888			1.70%
SNP_A-4282389		5.00%	3.50%
SNP_A-4284155			6.80%
SNP_A-4287059			3.40%
SNP_A-2167587			10.20%
SNP_A-2176547	3.30%	5.00%	
SNP_A-2243547			5.10%
SNP_A-2261675			5.10%
SNP_A-2262511	14.00%	11.70%	19.00%
SNP_A-4194811			5.10%
SNP_A-4245180	3.30%		
SNP_A-1869837			5.10%
SNP_A-1889187	5.00%	5.10%	2.40%
SNP_A-1934646		5.00%	13.60%
SNP_A-8364372		5.00%	3.40%
SNP_A-8464148			5.10%
SNP_A-8534276		20.00%	
SNP_A-8583319			13.60%
SNP_A-8605593			3.40%
SNP_A-8646911			5.10%
SNP_A-8683564			5.30%
SNP_A-8682132		5.00%	
SNP_A-8299547		1.70%	
SNP_A-8310429	1.70%	16.70%	3.40%
SNP_A-8405350		5.00%	1.70%
SNP_A-8397613	3.30%		1.70%
SNP_A-8426992		3.30%	
SNP_A-8442072			5.10%
SNP_A-8504767	1.70%		1.70%
SNP_A-8515850	16.70%		
