# Supplementary material for: Discovery of novel variants in genotyping arrays improves genotype retention and reduces ascertainment bias
Source: BMC Genomics. 2012 Jan 19;13:34. doi: 10.1186/1471-2164-13-34 (PMC3305361; doi:10.1186/1471-2164-13-34)
Supplement: Additional file 14 — Summary of unaligned probe sets. Probe-set sequences were aligned to the imputed genomes for each of 14 Sanger strains using BWA. The fraction of probe non-aligning probe sets is shown. Well-performing probe sets are those included in the present study, while excluded probe sets were removed due to poor performance across the 351 samples in this study. Excluded probe sets are an order of magnitude more likely to be non-aligning. [file 1471-2164-13-34-S14.PDF]

Table S9. Summary of unaligned probe sets.

	Well-Performing Probesets (N = 549,665)				Excluded Probesets (N = 73,458)			
Strain	At Least One Strand Unaligned	%	Both Strands Unaligned	%	At Least One Strand Unaligned	%	Both Strands Unaligned	%
**C57BL/6J**	3,378	0.31%	2,195	0.40%	3,836	2.61%	2,767	3.77%
**C57BL/6N**	3,402	0.31%	2,222	0.40%	3,838	2.61%	2,768	3.77%
**BALB/cJ**	4,029	0.37%	2,602	0.47%	4,417	3.01%	3,232	4.40%
**AKR**	4,097	0.37%	2,640	0.48%	4,440	3.02%	3,246	4.42%
**DBA**	4,086	0.37%	2,624	0.48%	4,467	3.04%	3,266	4.45%
**C3H**	4,082	0.37%	2,622	0.48%	4,471	3.04%	3,274	4.46%
**129/S1**	4,134	0.38%	2,664	0.48%	4,507	3.07%	3,307	4.50%
**CBA**	4,142	0.38%	2,684	0.49%	4,524	3.08%	3,311	4.51%
**A/J**	4,138	0.38%	2,675	0.49%	4,530	3.08%	3,324	4.53%
**NOD**	4,174	0.38%	2,686	0.49%	4,634	3.15%	3,389	4.61%
**LP/J**	4,259	0.39%	2,723	0.50%	4,622	3.15%	3,387	4.61%
**NZO**	4,393	0.40%	2,814	0.51%	4,751	3.23%	3,489	4.75%
**WSB/EiJ**	5,128	0.47%	3,351	0.61%	5,132	3.49%	3,764	5.12%
**PWK**	9,046	0.82%	5,747	1.05%	8,523	5.80%	6,321	8.60%
**CAST/EiJ**	12,043	1.10%	8,028	1.46%	8,691	5.92%	6,520	8.88%
	74,531	0.45%	48,277	0.59%	75,383	3.42%	55,365	5.02%
